# Crystal structures of two new six-coordinate iron(III) complexes with 1,2-bis(diphenyl­phosphane) ligands

**DOI:** 10.1107/S2056989018006898

**Published:** 2018-05-15

**Authors:** Derek L. McNeil, Daihlia J. Beckford, Jared L. Kneebone, Stephanie H. Carpenter, William W. Brennessel, Michael L. Neidig

**Affiliations:** a Department of Chemistry, University of Rochester, Rochester, NY 14627, USA

**Keywords:** iron, bidentate phosphane, inversion twin, crystal structure, iron-catalysed cross-coupling

## Abstract

Structural characterization of the ionic complexes [FeCl_2_(C_26_H_22_P_2_)_2_][FeCl_4_]·0.59CH_2_Cl_2_ or [(dppen)_2_FeCl_2_][FeCl_4_]·0.59CH_2_Cl_2_ (dppen = *cis*-1,2-bis­(di­phenyl­phosphane)ethyl­ene, P_2_C_26_H_22_) and [FeCl_2_(C_30_H_24_P_2_)_2_][FeCl_4_]·CH_2_Cl_2_ or [(dpbz)_2_FeCl_2_][FeCl_4_]·CH_2_Cl_2_ (dpbz = 1,2-bis­(di­phenyl­phosphane)benzene, P_2_C_30_H_24_) demonstrates *trans* coordination of two bidentate phosphane ligands (bis­phosphanes) to a single iron(III) center, resulting in six-coordinate cationic complexes that are balanced in charge by tetra­chlorido­ferrate(III) monoanions. The *trans* bis­phosphane coordination is consistent will all previously reported mol­ecular structures of six coordinate iron(III) complex cations with a (PP)_2_ *X*_2_ (*X* = halido) donor set. The complex with dppen crystallizes in the centrosymmetric space group *C*2/*c* as a partial-occupancy [0.592 (4)] di­chloro­methane solvate, while the dpbz-ligated complex crystallizes in the triclinic space group *P*1 as a full di­chloro­methane monosolvate. Furthermore, the crystal studied of [(dpbz)_2_FeCl_2_][FeCl_4_]·CH_2_Cl_2_ was an inversion twin, whose component mass ratio refined to 0.76 (3):0.24 (3). Beyond a few very weak C—H⋯Cl and C—H⋯π inter­actions, there are no significant supra­molecular features in either structure.

## Abstract 2

Two new structures from a very small group to date of six-coordinate monocationic iron(III) complexes containing two bidentate phosphane and two halido ligands are presented as di­chloro­methane solvates. The Fe—P and Fe—Cl bond lengths are longer and shorter, respectively, than those previously reported for cations in this group.

## Chemical context   

Bidentate phosphanes (bis­phosphanes) are versatile supporting ligands in coordination chemistry because of the accessibility of various electronic and steric properties through manipulation of their backbone structures and phospho­rus substituents. While these ligands are commonly used to stabilize low-valent metal complexes due to their function as both σ-donor and π-acceptor ligands, bis­phosphane ligands have also been observed to support metal centers in higher oxidation states. For example, there exist a few structurally characterized examples of iron(III) complexes in which two bis­phosphane ligands are coordinated to a single metal center, resulting in axial coordination of halido (*X*) ligands. These complex cations have been shown to be accessible through a variety of synthetic routes (Higgins *et al.*, 1985 ▸; Higgins & Levason, 1985 ▸; Field *et al.*, 1990 ▸, 2000 ▸; Evans *et al.*, 1992 ▸; Miller *et al.*, 2002 ▸; Hoffert *et al.*, 2011 ▸). A review of the literature finds that investigations into these complexes date back almost sixty years to the work of Chatt and Hayter, in which three distinct iron(III) bis­phosphane complexes, formulated as complex salts with the mol­ecular structures [(PP)_2_FeCl_2_][FeCl_4_] [PP = 1,2-bis­(di­ethyl­phosphano)benzene (debz), 1,2-bis­(di­ethyl­phosphano)ethane (depe), and 1,2-bis­(di­methyl­phosphano)ethane (dmpe)], were prepared through the reaction of iron(III) chloride with one shoichiometric equivalent of bis­phosphane (Chatt & Hayter, 1961 ▸; for later reports of various preparative methods of similar compounds, see: Levason *et al.*, 1975 ▸; Gargano *et al.*, 1975 ▸; Warren *et al.*, 1976 ▸; Higgins & Levason, 1985 ▸). Structural confirmation for this general mol­ecular formula was achieved through the crystallographic characterization of [(dmpe)_2_FeCl_2_][FeCl_4_], although this synthesis employed photolytic oxidation of the iron(II) complex (dmpe)_2_FeCl_2_ and not direct reaction of an iron(III) precursor with bis­phosphane (Field *et al.*, 1990 ▸). At the time of this report, the only other known mol­ecular structure for a six-coordinate iron(III) complex cation bearing a (PP)_2_ *X*_2_ ligand set was [(*o*-C_6_F_4_(PMe_2_)_2_)_2_FeCl_2_][BF_4_] (Higgins *et al.*, 1985 ▸; Higgins & Levason, 1985 ▸). This particular species was synthesized through metathesis of the original tetra­chlorido­ferrate(III) anion with HBF_4_. The initial salt, [(*o*-C_6_F_4_(PMe_2_)_2_)_2_FeCl_2_][FeCl_4_], prepared *via* a nearly 1:1 stoichio­metric reaction of iron(III) chloride with *o*-C_6_F_4_(PMe_2_)_2_, was not structurally characterized.
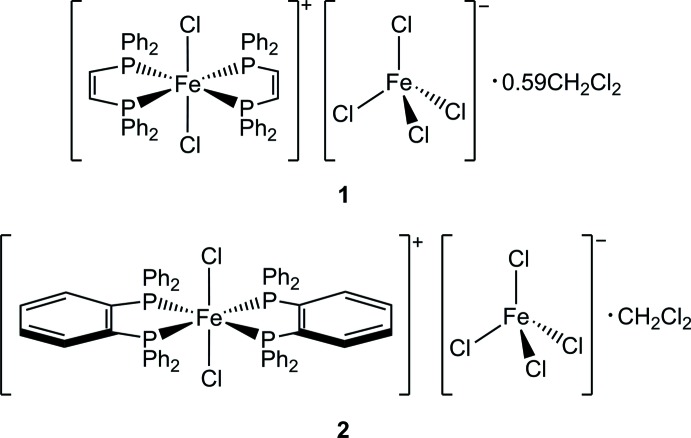


Our group is inter­ested in the application of bis­phosphanes as supporting ligands within iron-catalyzed cross-coupling reactions. Numerous literature protocols for iron-catalyzed cross-coupling methods involve use of bis­phosphanes as substoichiometric additives in conjunction with iron(II) or iron(III) salts, promoting the formation of the active catalyst *in situ*. Methods development in our laboratory using the dppen ligand in conjunction with iron(III) chloride resulted in the formation of [(dppen)_2_FeCl_2_][FeCl_4_] (**1**) from reaction mixtures and its subsequent structural characterization. As reported herein, **1** was then independently prepared *via* the method of Chatt & Hayter (1961 ▸). While we have not observed this compound to exhibit catalytic effectiveness in cross-coupling, a literature search indicated that this ionic complex was first synthesized in the 1970s using the same reaction stoichiometry (Levason *et al.*, 1975 ▸). This report lacked structural characterization of the complex, but its formulation as a complex salt was supported by magnetic susceptibility, Mössbauer, and IR absorption measurements. Upon confirming the structure of **1**, we sought to examine an analogous species, [(dpbz)_2_FeCl_2_][FeCl_4_] (**2**), by taking advantage of the same steric substitution at phospho­rus, but with a slightly varied backbone character (*ortho*-phenyl­ene in place of the C_2_H_2_ of dppen). Such studies are important as they expand the coordination chemistry library of iron(III) complexes bearing bis­phosphane ligands. In addition, **1** and **2** join only two other structurally characterized examples of coordinatively saturated iron(III) monocations with a (PP)_2_ *X*_2_ ligand set that have been synthesized without using oxidative methods (Miller *et al.*, 2002 ▸, Higgins & Levason, 1985 ▸).

## Structural commentary   

**Figure 1:**
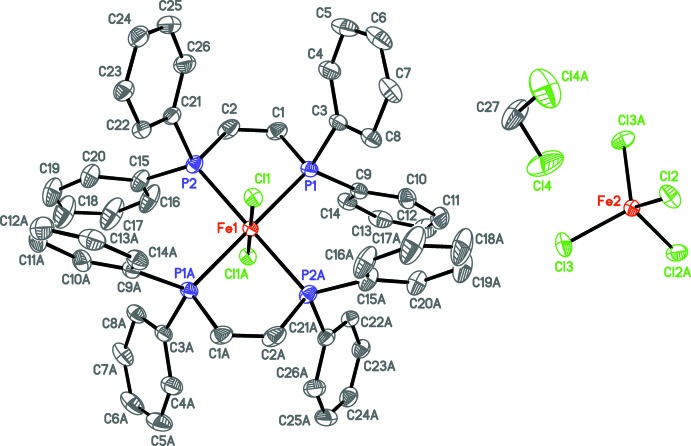
Displacement ellipsoid plot of **1** drawn at the 50% probability level with hydrogen atoms omitted. The full cation of the title formula is generated by a crystallographic inversion center (1 − *x*, 1 − *y*, 1 − *z*) at atom Fe1. The full anion is generated by a crystallographic twofold axis (−*x*, *y*, 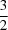 − *z*), which includes atom Fe2. The symmetry-equivalent atoms of the di­chloro­methane solvent mol­ecule are generated by a crystallographic twofold axis (1 − *x*, *y*, 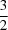 − *z*) that contains atom C27.

Both **1** and **2** are characterized as six-coordinate complex cations in which the iron(III) center is ligated by two bis­phosphane ligands (dppen in **1**, dpbz in **2**) in a *trans* fashion (see Scheme). The two retained chlorido ligands are thus coordinated axially, and the displaced chlorido ligand results in generation of a single tetra­chlorido­ferrate(III) anion in both cases. Compound **1** (Fig. 1 ▸) crystallizes in the centrosymmetric space group *C*2/*c*. The iron atom of the cation is located at a crystallographic inversion center, resulting in Cl—Fe—Cl and *trans* P—Fe—P angles of 180°. Deviation from ideal octa­hedral geometry is due to the 80.92 (2)° bite angles of the P—Fe—P chelate rings (Table 1 ▸). The Fe—P distances are considerably longer than those of the other structurally characterized iron(III) cations with a (PP)_2_ *X*_2_ donor set (range 2.29–2.34 Å; Groom *et al.*, 2016 ▸, see *Database survey* below), but with shorter Fe—Cl distances than those of the other reported *X* = Cl compounds (range 2.23–2.25 Å). The ethyl­ene backbones of each dppen ligand in the cation of **1** are bent out of the equatorial plane by 24.86 (8)°. The tetra­chlorido­ferrate(III) anion lies along a crystallographic twofold axis that includes the metal center. Phenyl group C3—C8 (and thus its symmetry equivalent, Fig. 1 ▸) is modeled as disordered over two positions [0.561 (6):0.439 (6)].

**Figure 2:**
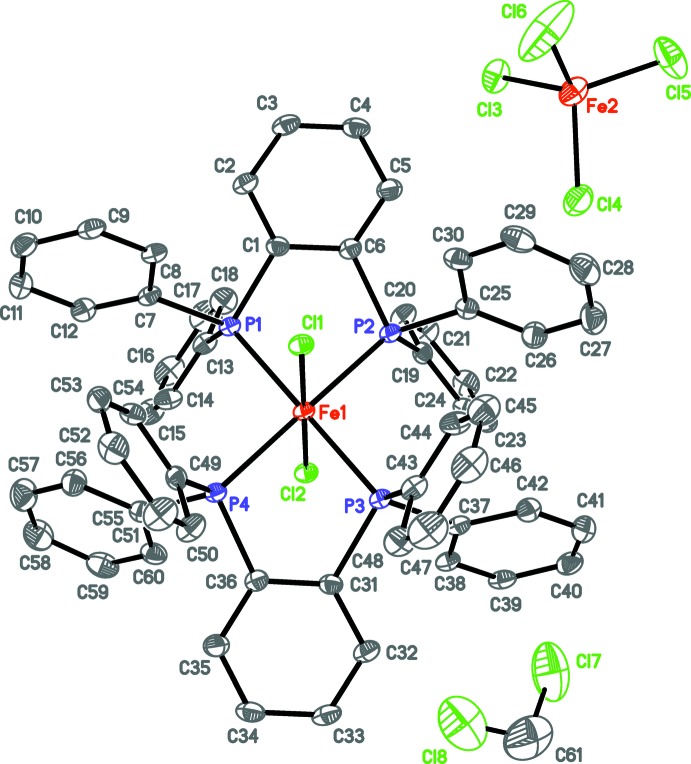
Displacement ellipsoid plot of **2** drawn at the 50% probability level with hydrogen atoms omitted. Only the major component of disorder of the di­chloro­methane solvent mol­ecule is shown.

The asymmetric unit of **2** contains the cation, anion, and solvent mol­ecule in general positions. The solvent mol­ecule was modeled as disordered over three positions [0.740 (3):0.136 (3):0.124 (3)]. Despite the structural similarity of the backbone linkers and steric periphery of dppen and dpbz, the space group assignment and crystallographic symmetry of **2** contrasted from **1**. Metrically, however, **1** and **2** are quite similar. The axial chlorido ligands within the cation of **2** are located at Fe—Cl distances very close to that found in the cation of **1** and the Cl—Fe—Cl and *trans* P—Fe—P angles are very nearly linear (Fig. 2 ▸, Table 2 ▸). Additionally, the bite angles in the cation of **2** as well as Fe—Cl distances and Cl—Fe—Cl angles of its tetra­chlorido­ferrate(III) anion are very similar to those of **1**. As observed for the ethyl­ene backbones of the dppen ligands of **1**, the *ortho*-phenyl­ene backbones of the dpbz ligands in **2** are also canted out the equatorial plane by 21.9 (1) and 22.9 (1)°. The crystal of **2** studied was an inversion twin, whose component mass ratio refined to 0.76 (3):0.24 (3).

Both **1** and **2** are di­chloro­methane solvates under the common crystallization procedure used (see below). In **1**, the solvent mol­ecule is located along a crystallographic twofold axis that includes the carbon atom. Crystal desolvation is suspected, since its occupancy only refined to 0.592 (4). In contrast, **2** was found to possess full occupation of co-crystallized di­chloro­methane, modeled as disordered over three general positions [0.740 (3):0.136 (3):0.124 (3)].

## Supra­molecular features   

There are no significant supra­molecular features beyond a few very weak C—H⋯Cl and C—H⋯π inter­actions.

## Database survey   

Outside of **1** and **2** reported herein, there are eight examples of ionic iron(III) compounds bearing *trans*-coordinating bis­phosphane ligands in an overall (PP)_2_ *A*_2_ (*A* = formally monoanionic ligand) environment reported in the Cambridge Structural Database (CSD, Version 5.39, update No. 2, February 2018; Groom *et al.*, 2016 ▸). The axial *A* ligands of the cations include two chlorido (CSD refcode DABCEO, Higgins *et al.*, 1985 ▸; recode VOBHUP, Field *et al.*, 1990 ▸; recode XAZZIH, Field *et al.*, 2000 ▸; refcode BAJLAA, Miller *et al.*, 2002 ▸), bromido and hydrido (refcode PABSUG; Evans *et al.*, 1992 ▸), and chlorido and alkynyl (refcodes NAWMIJ, NAWMOP, NAWMUV; Hoffert *et al.*, 2011 ▸). These structures include a variety of counter-anions: [FeCl_4_]^−^, [BF_4_]^−^, [BPh_4_]^−^, [Cl]^−^, [SO_3_CF_3_]^−^, and [B(3,5-CF_3_Ph)_4_]^−^. Only one of these examples, *trans*-[(*o*-C_6_F_4_(PMe_2_)_2_)_2_FeCl_2_][BF_4_], contains a bis­phosphane ligand with an unsaturated backbone linker (Higgins *et al.*, 1985 ▸). Just as in **1** and **2**, the fluoro-substituted *ortho*-phenyl­ene backbone of *trans*-[(*o*-C_6_F_4_(PMe_2_)_2_)_2_­FeCl_2_][BF_4_] is also bent out of from the FeP_4_ equatorial plane (17.6 °).

## Synthesis and crystallization   

Anhydrous FeCl_3_ (98%, Alfa Aesar), *cis*-1,2-bis­(di­phenyl­phosphanel)ethyl­ene (dppen, 98%, Strem), and 1,2-bis­(di­phenyl­phosphane)benzene (dpbz, 98%, Strem) were used in the synthesis of **1** and **2** without further purification. The syntheses of both compounds were performed under a di­nitro­gen atmosphere in a drybox. 80 mg FeCl_3_ (0.49 mmol) was dissolved in 5 ml THF (Aldrich, anhydrous, 99.9%, inhibitor-free), resulting in a yellow–green solution. In a separate vial, 200 mg dppen (or 225 mg dpbz for **2**, 0.50 mmol in either case) was dissolved in 10 ml THF. At room temperature, the solution of bis­phosphane was added to the stirring solution of FeCl_3_, resulting in immediate formation of a dark green precipitate in both cases. Each reaction was stirred for 5 min following complete addition of the bis­phosphane solution, filtered, and the resulting green solid was dried under vacuum. In both cases, analytically pure microcrystalline solid was isolated in nearly qu­anti­tative yield. [FeCl_2_(dppen)_2_][FeCl_4_]: Yield: 94%. Elemental analysis: calculated: 55.903 C, 3.970 H; found: 56.327 C, 4.342 H. [FeCl_2_(dpbz)_2_][FeCl_4_]: Yield: 89%. Elemental analysis: calculated: 59.199 C, 3.974 H; found: 59.526 C, 4.452.

Once isolated and dried, solid **1** and **2** were found to be indefinitely stable outside of an inert atmosphere (greater than one year). Both complexes were crystallized by layering toluene over a concentrated di­chloro­methane solution of the complex and allowing the layers to diffuse at room temperature (anhydrous solvents were not used during crystallizations). Red–green dichroic single crystals suitable for X-ray diffraction studies were generally observed to crystallize within 24 h. Out of a large number of polar and non-polar common organic solvents examined, only di­chloro­methane, chloro­form, acetone, and nitro­methane appreciably solubilized **1** and **2**. During preparation of crystallizations, di­chloro­methane solutions of **1** and **2** were observed under incandescent light to be green at low concentrations and red at high concentrations.

## Refinement   

Crystal data, data collection and structure refinement details are summarized in Table 3 ▸.

Phenyl ring C3–C8 of **1** was modeled as disordered over two general positions [0.561 (6):0.439 (6)]. Analogous bond lengths and angles between the two positions and in both directions around the rings were restrained to be similar. Additionally the P1—C3 and P1—C3′ bond lengths were restrained to be similar. Anisotropic displacement parameters for pairs of proximal atoms were constrained to be equivalent. The occupancy of the cocrystallized di­chloro­methane solvent mol­ecule refined to 0.592 (4), which is consistent with crystal desolvation.

**2** was refined as an inversion twin in *P*1 whose twin component mass ratio refined to 0.76 (3):0.24 (3). Because of significant parameter correlation, anisotropic displacement parameters for pseudosymmetrically related atom pairs were constrained to be equivalent. The co-crystallized di­chloro­methane solvent mol­ecule is modeled as disordered over three positions [0.740 (3):0.136 (3):0.124 (3)]. Analogous bond lengths and angles among the three positions of the disordered di­chloro­methane solvent mol­ecule were restrained to be similar. Anisotropic displacement parameters for proximal and pseudosymmetrically related atoms were constrained to be equivalent.

A solution and refinement of **2** in centrosymmetric space group *P* 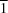 caused an increase in the *R*1 residual (strong data) from 0.071 to 0.118, which was not unexpected given the uneven twin component mass ratio when refined in *P*1. In the centrosymmetric model, the anion and solvent were modeled pairwise as disordered over a crystallographic inversion center.

H atoms were given riding models: aromatic/*sp* ^2^, C—H = 0.95 Å, and methyl­ene, C—H = 0.99 Å, with *U*_iso_(H) = 1.2*U*_eq_(C).

For **1** the maximum residual peak of 0.74 e^−^ Å^−3^ and the deepest hole of −0.67 e^−^ Å^−3^ are found 0.72 and 0.82 Å, respectively, from atom CL4.

For **2** the maximum residual peak of 1.23 e^−^ Å^−3^ and the deepest hole of −1.39 e^−^ Å^−3^ are found 0.22 and 0.05 Å from atoms CL5 and C61 of the disordered solvent molcule, respectively.

## Supplementary Material

Crystal structure: contains datablock(s) 1, 2, global. DOI: 10.1107/S2056989018006898/pj2052sup1.cif

Structure factors: contains datablock(s) 1. DOI: 10.1107/S2056989018006898/pj20521sup2.hkl

Structure factors: contains datablock(s) 2. DOI: 10.1107/S2056989018006898/pj20522sup3.hkl

CCDC references: 1841465, 1841464

Additional supporting information:  crystallographic information; 3D view; checkCIF report

## Figures and Tables

**Table 1:** Selected geometric parameters (Å, °) for **1** [Chem scheme1]

Fe1—Cl1	2.2135 (6)	Fe1—P2	2.3738 (6)
Fe1—P1	2.3662 (6)		
			
Cl1^i^—Fe1—Cl1	180.0	Cl1—Fe1—P2^i^	87.58 (2)
Cl1—Fe1—P1	92.38 (2)	Cl1—Fe1—P2	92.42 (2)
Cl1—Fe1—P1^i^	87.62 (2)	P1—Fe1—P2	80.92 (2)
P1—Fe1—P1^i^	180.0	P2^i^—Fe1—P2	180.0

Symmetry code: (i) 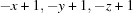.

**Table 2:** Selected geometric parameters (Å, °) for **2** [Chem scheme1]

Fe1—Cl2	2.218 (2)	Fe1—P2	2.376 (2)
Fe1—Cl1	2.223 (2)	Fe1—P4	2.377 (2)
Fe1—P3	2.374 (2)	Fe1—P1	2.388 (2)
			
Cl2—Fe1—Cl1	179.87 (12)	P3—Fe1—P4	80.75 (8)
Cl2—Fe1—P3	87.69 (8)	P2—Fe1—P4	179.33 (10)
Cl1—Fe1—P3	92.26 (8)	Cl2—Fe1—P1	92.05 (8)
Cl2—Fe1—P2	92.82 (8)	Cl1—Fe1—P1	87.99 (7)
Cl1—Fe1—P2	87.30 (8)	P3—Fe1—P1	179.74 (10)
P3—Fe1—P2	98.58 (8)	P2—Fe1—P1	81.38 (8)
Cl2—Fe1—P4	87.23 (8)	P4—Fe1—P1	99.29 (8)
Cl1—Fe1—P4	92.65 (8)		

**Table 3:** Experimental details

	**1**	**2**
Crystal data		
Chemical formula	[FeCl_2_(C_26_H_22_P_2_)_2_][FeCl_4_]·0.59CH_2_Cl_2_	[FeCl_2_(C_30_H_24_P_2_)_2_][FeCl_4_]·CH_2_Cl_2_
*M*_r_	1167.47	1302.19
Crystal system, space group	Monoclinic, *C*2/*c*	Triclinic, *P*1
Temperature (K)	100	100
*a*, *b*, *c* (Å)	9.7528 (7), 23.6871 (17), 23.6871 (17)	9.8771 (7), 12.6516 (8), 12.8258 (8)
α, β, γ (°)	90, 100.541 (2), 90	81.058 (1), 83.050 (1), 68.335 (1)
*V* (Å^3^)	5379.7 (7)	1467.87 (17)
*Z*	4	1
Radiation type	Mo *K*α	Mo *K*α
μ (mm^−1^)	1.05	1.01
Crystal size (mm)	0.22 × 0.22 × 0.10	0.24 × 0.20 × 0.08

Data collection		
Diffractometer	Bruker SMART APEX II CCD platform	Bruker SMART APEX II CCD platform
Absorption correction	Multi-scan (*SADABS*; Krause *et al.*, 2015 ▸)	Multi-scan (*SADABS*; Krause *et al.*, 2015 ▸)
*T*_min_, *T*_max_	0.643, 0.746	0.644, 0.747
No. of measured, independent and observed [*I* > 2σ(*I*)] reflections	71735, 8962, 6273	27211, 22268, 11890
*R*_int_	0.091	0.050
(sin θ/λ)_max_ (Å^−1^)	0.736	0.807

Refinement		
*R*[*F* ^2^ > 2σ(*F* ^2^)], *wR*(*F* ^2^), *S*	0.048, 0.119, 1.05	0.073, 0.182, 1.00
No. of reflections	8962	22268
No. of parameters	325	488
No. of restraints	33	34
H-atom treatment	H-atom parameters constrained	H-atom parameters constrained
Δρ_max_, Δρ_min_ (e Å^−3^)	0.74, −0.67	1.23, −1.39
Absolute structure	–	Refined as an inversion twin.
Absolute structure parameter	–	0.24 (3)

Computer programs: *APEX3* (Bruker, 2016 ▸), *SAINT* (Bruker, 2013 ▸), *SHELXT2014*/5 (Sheldrick, 2015*a* ▸), *SHELXL2016*/6 (Sheldrick, 2015*b* ▸), *SHELXL2018*/3 (Sheldrick, 2015*b* ▸), *SHELXTL* (Sheldrick, 2008 ▸).

## supplementary crystallographic information

### trans-Bis[1,2-bis(diphenylphosphane)ethylene]dichloridoiron(III) tetrachloridoferrate(III) dichloromethane 0.59-solvate (1) Crystal data

**Table:**

[FeCl_2_(C_26_H_22_P_2_)_2_][FeCl_4_]·0.59CH_2_Cl_2_	*F*(000) = 2380
*M**_r_* = 1167.47	*D*_x_ = 1.441 Mg m^−^^3^
Monoclinic, *C*2/*c*	Mo *K*α radiation, λ = 0.71073 Å
*a* = 9.7528 (7) Å	Cell parameters from 4040 reflections
*b* = 23.6871 (17) Å	θ = 2.3–29.5°
*c* = 23.6871 (17) Å	µ = 1.05 mm^−^^1^
β = 100.541 (2)°	*T* = 100 K
*V* = 5379.7 (7) Å^3^	Plate, red-violet
*Z* = 4	0.22 × 0.22 × 0.10 mm

### trans-Bis[1,2-bis(diphenylphosphane)ethylene]dichloridoiron(III) tetrachloridoferrate(III) dichloromethane 0.59-solvate (1) Data collection

**Table:**

Bruker SMART APEX II CCD platform diffractometer	6273 reflections with *I* > 2σ(*I*)
Radiation source: fine-focus sealed tube	*R*_int_ = 0.091
ω scans	θ_max_ = 31.5°, θ_min_ = 1.7°
Absorption correction: multi-scan (SADABS; Krause *et al*., 2015)	*h* = −14→14
*T*_min_ = 0.643, *T*_max_ = 0.746	*k* = −34→34
71735 measured reflections	*l* = −34→34
8962 independent reflections	

### trans-Bis[1,2-bis(diphenylphosphane)ethylene]dichloridoiron(III) tetrachloridoferrate(III) dichloromethane 0.59-solvate (1) Refinement

**Table:**

Refinement on *F*^2^	Primary atom site location: dual
Least-squares matrix: full	Secondary atom site location: difference Fourier map
*R*[*F*^2^ > 2σ(*F*^2^)] = 0.048	Hydrogen site location: inferred from neighbouring sites
*wR*(*F*^2^) = 0.119	H-atom parameters constrained
*S* = 1.05	*w* = 1/[σ^2^(*F*_o_^2^) + (0.0418*P*)^2^ + 11.3134*P*] where *P* = (*F*_o_^2^ + 2*F*_c_^2^)/3
8962 reflections	(Δ/σ)_max_ = 0.001
325 parameters	Δρ_max_ = 0.74 e Å^−^^3^
33 restraints	Δρ_min_ = −0.67 e Å^−^^3^

### trans-Bis[1,2-bis(diphenylphosphane)ethylene]dichloridoiron(III) tetrachloridoferrate(III) dichloromethane 0.59-solvate (1) Special details

**Table:**

Geometry. All esds (except the esd in the dihedral angle between two l.s. planes) are estimated using the full covariance matrix. The cell esds are taken into account individually in the estimation of esds in distances, angles and torsion angles; correlations between esds in cell parameters are only used when they are defined by crystal symmetry. An approximate (isotropic) treatment of cell esds is used for estimating esds involving l.s. planes.
Refinement. Phenyl ring C3-C8 is modeled as disordered over two positions (56:44). Analogous bond lengths and angles between the two positions were restrained to be similar. Anisotropic displacement parameters for pairs of proximal atoms were constrained to be equivalent. The occupancy of the cocrystallized dichloromethane solvent molecule refined to 0.592 (4).

### trans-Bis[1,2-bis(diphenylphosphane)ethylene]dichloridoiron(III) tetrachloridoferrate(III) dichloromethane 0.59-solvate (1) Fractional atomic coordinates and isotropic or equivalent isotropic displacement parameters (Å^2^)

**Table:**

	*x*	*y*	*z*	*U*_iso_*/*U*_eq_	Occ. (<1)
Fe1	0.500000	0.500000	0.500000	0.01605 (10)	
Cl1	0.71798 (6)	0.53023 (2)	0.52215 (2)	0.02045 (12)	
P1	0.46240 (6)	0.51308 (3)	0.59502 (3)	0.01981 (13)	
P2	0.56127 (7)	0.40841 (3)	0.53686 (3)	0.02134 (13)	
C1	0.4343 (3)	0.44397 (12)	0.62312 (12)	0.0295 (6)	
H1	0.390120	0.440031	0.655474	0.035*	
C2	0.4761 (3)	0.39907 (12)	0.59802 (13)	0.0321 (6)	
H2	0.461628	0.362302	0.611842	0.038*	
C3	0.605 (2)	0.5342 (6)	0.6520 (8)	0.0227 (16)	0.439 (6)
C4	0.6555 (15)	0.4985 (4)	0.6979 (5)	0.0311 (15)	0.439 (6)
H4	0.618358	0.461673	0.699969	0.037*	0.439 (6)
C5	0.7618 (11)	0.5184 (4)	0.7406 (4)	0.0393 (15)	0.439 (6)
H5	0.798729	0.494642	0.772091	0.047*	0.439 (6)
C6	0.8136 (10)	0.5718 (4)	0.7377 (4)	0.0386 (16)	0.439 (6)
H6	0.885234	0.584895	0.767556	0.046*	0.439 (6)
C7	0.7637 (10)	0.6067 (4)	0.6925 (4)	0.0322 (13)	0.439 (6)
H7	0.801045	0.643578	0.690908	0.039*	0.439 (6)
C8	0.6576 (15)	0.5879 (5)	0.6487 (6)	0.0258 (14)	0.439 (6)
H8	0.622193	0.611721	0.617137	0.031*	0.439 (6)
C3'	0.5987 (16)	0.5479 (4)	0.6468 (6)	0.0227 (16)	0.561 (6)
C4'	0.6649 (11)	0.5188 (3)	0.6964 (4)	0.0311 (15)	0.561 (6)
H4'	0.640148	0.480827	0.702639	0.037*	0.561 (6)
C5'	0.7671 (8)	0.5460 (3)	0.7365 (3)	0.0393 (15)	0.561 (6)
H5'	0.810045	0.526376	0.770080	0.047*	0.561 (6)
C6'	0.8060 (7)	0.6004 (3)	0.7280 (3)	0.0386 (16)	0.561 (6)
H6'	0.875271	0.618286	0.755653	0.046*	0.561 (6)
C7'	0.7444 (7)	0.6293 (3)	0.6793 (3)	0.0322 (13)	0.561 (6)
H7'	0.771181	0.667151	0.673465	0.039*	0.561 (6)
C8'	0.6426 (11)	0.6029 (3)	0.6384 (4)	0.0258 (14)	0.561 (6)
H8'	0.602754	0.622673	0.604511	0.031*	0.561 (6)
C9	0.3051 (2)	0.54964 (11)	0.60637 (10)	0.0213 (5)	
C10	0.3035 (3)	0.60629 (12)	0.62219 (12)	0.0283 (5)	
H10	0.388476	0.626830	0.630628	0.034*	
C11	0.1786 (3)	0.63282 (14)	0.62566 (13)	0.0377 (7)	
H11	0.177872	0.671691	0.635490	0.045*	
C12	0.0553 (3)	0.60285 (15)	0.61486 (13)	0.0404 (8)	
H12	−0.030285	0.621444	0.616380	0.048*	
C13	0.0553 (3)	0.54595 (14)	0.60185 (12)	0.0338 (6)	
H13	−0.029162	0.524979	0.596454	0.041*	
C14	0.1798 (2)	0.51970 (12)	0.59672 (11)	0.0249 (5)	
H14	0.179670	0.480912	0.586525	0.030*	
C15	0.4992 (3)	0.34577 (11)	0.49472 (13)	0.0292 (6)	
C16	0.3571 (3)	0.33373 (12)	0.48605 (16)	0.0409 (8)	
H16	0.296816	0.356038	0.504190	0.049*	
C17	0.3039 (4)	0.28904 (13)	0.4508 (2)	0.0542 (10)	
H17	0.206782	0.281126	0.444628	0.065*	
C18	0.3916 (4)	0.25578 (13)	0.4246 (2)	0.0554 (10)	
H18	0.354057	0.226315	0.399149	0.066*	
C19	0.5340 (3)	0.26585 (13)	0.43563 (17)	0.0434 (8)	
H19	0.594411	0.241985	0.419199	0.052*	
C20	0.5891 (3)	0.31054 (11)	0.47053 (13)	0.0323 (6)	
H20	0.686832	0.317231	0.477997	0.039*	
C21	0.7448 (3)	0.39546 (10)	0.56642 (11)	0.0235 (5)	
C22	0.8443 (3)	0.40043 (10)	0.53138 (11)	0.0230 (5)	
H22	0.815723	0.409121	0.491821	0.028*	
C23	0.9851 (3)	0.39282 (11)	0.55371 (12)	0.0270 (5)	
H23	1.052194	0.395727	0.529381	0.032*	
C24	1.0276 (3)	0.38094 (11)	0.61176 (13)	0.0311 (6)	
H24	1.123860	0.376665	0.627434	0.037*	
C25	0.9290 (3)	0.37537 (14)	0.64658 (13)	0.0383 (7)	
H25	0.957818	0.366739	0.686137	0.046*	
C26	0.7883 (3)	0.38229 (13)	0.62420 (12)	0.0345 (6)	
H26	0.721343	0.378035	0.648409	0.041*	
Fe2	0.000000	0.80718 (2)	0.750000	0.02335 (12)	
Cl2	0.18469 (8)	0.86224 (3)	0.76337 (3)	0.03463 (16)	
Cl3	−0.00845 (8)	0.75564 (3)	0.67250 (3)	0.03743 (17)	
C27	0.500000	0.6989 (3)	0.750000	0.055 (3)	0.592 (4)
H27A	0.480195	0.674166	0.781219	0.066*	0.2960 (19)
H27B	0.519802	0.674164	0.718782	0.066*	0.2960 (19)
Cl4	0.35377 (18)	0.73688 (9)	0.72448 (9)	0.0700 (8)	0.592 (4)

### trans-Bis[1,2-bis(diphenylphosphane)ethylene]dichloridoiron(III) tetrachloridoferrate(III) dichloromethane 0.59-solvate (1) Atomic displacement parameters (Å^2^)

**Table:**

	*U*^11^	*U*^22^	*U*^33^	*U*^12^	*U*^13^	*U*^23^
Fe1	0.0147 (2)	0.0175 (2)	0.0161 (2)	−0.00051 (16)	0.00312 (16)	−0.00011 (17)
Cl1	0.0159 (2)	0.0237 (3)	0.0217 (3)	−0.0022 (2)	0.00337 (19)	−0.0017 (2)
P1	0.0166 (3)	0.0264 (3)	0.0167 (3)	0.0014 (2)	0.0037 (2)	0.0001 (2)
P2	0.0204 (3)	0.0198 (3)	0.0252 (3)	0.0025 (2)	0.0079 (2)	0.0039 (2)
C1	0.0302 (13)	0.0347 (14)	0.0263 (13)	0.0083 (11)	0.0126 (11)	0.0114 (11)
C2	0.0319 (14)	0.0316 (14)	0.0368 (15)	0.0083 (11)	0.0173 (12)	0.0163 (12)
C3	0.0173 (17)	0.034 (5)	0.016 (3)	0.006 (4)	0.0023 (13)	−0.002 (3)
C4	0.031 (2)	0.040 (5)	0.0219 (15)	0.002 (4)	0.0027 (14)	0.000 (3)
C5	0.034 (2)	0.058 (5)	0.0226 (19)	0.000 (4)	−0.0047 (16)	0.002 (4)
C6	0.0271 (19)	0.065 (5)	0.023 (3)	−0.007 (4)	0.0028 (18)	−0.012 (3)
C7	0.026 (2)	0.046 (4)	0.026 (3)	−0.011 (3)	0.009 (2)	−0.011 (3)
C8	0.021 (2)	0.036 (4)	0.020 (3)	−0.002 (3)	0.0026 (19)	−0.004 (3)
C3'	0.0173 (17)	0.034 (5)	0.016 (3)	0.006 (4)	0.0023 (13)	−0.002 (3)
C4'	0.031 (2)	0.040 (5)	0.0219 (15)	0.002 (4)	0.0027 (14)	0.000 (3)
C5'	0.034 (2)	0.058 (5)	0.0226 (19)	0.000 (4)	−0.0047 (16)	0.002 (4)
C6'	0.0271 (19)	0.065 (5)	0.023 (3)	−0.007 (4)	0.0028 (18)	−0.012 (3)
C7'	0.026 (2)	0.046 (4)	0.026 (3)	−0.011 (3)	0.009 (2)	−0.011 (3)
C8'	0.021 (2)	0.036 (4)	0.020 (3)	−0.002 (3)	0.0026 (19)	−0.004 (3)
C9	0.0175 (10)	0.0288 (12)	0.0181 (11)	0.0019 (9)	0.0044 (8)	−0.0001 (9)
C10	0.0304 (13)	0.0308 (13)	0.0259 (13)	0.0005 (11)	0.0108 (10)	−0.0028 (11)
C11	0.0471 (18)	0.0383 (16)	0.0328 (16)	0.0147 (14)	0.0204 (13)	0.0000 (12)
C12	0.0314 (15)	0.060 (2)	0.0336 (16)	0.0217 (14)	0.0166 (12)	0.0098 (15)
C13	0.0194 (12)	0.0573 (19)	0.0249 (13)	0.0028 (12)	0.0049 (10)	0.0055 (13)
C14	0.0197 (11)	0.0357 (14)	0.0200 (12)	−0.0018 (10)	0.0056 (9)	−0.0004 (10)
C15	0.0264 (12)	0.0180 (11)	0.0436 (16)	0.0006 (10)	0.0076 (11)	0.0021 (11)
C16	0.0257 (13)	0.0199 (13)	0.079 (2)	−0.0008 (11)	0.0139 (15)	0.0006 (14)
C17	0.0311 (16)	0.0241 (15)	0.106 (3)	−0.0062 (12)	0.0070 (18)	−0.0078 (18)
C18	0.0420 (18)	0.0233 (15)	0.097 (3)	−0.0058 (13)	0.0033 (19)	−0.0179 (17)
C19	0.0376 (16)	0.0255 (14)	0.067 (2)	0.0009 (12)	0.0100 (16)	−0.0107 (14)
C20	0.0289 (13)	0.0230 (13)	0.0455 (17)	0.0009 (10)	0.0083 (12)	−0.0008 (12)
C21	0.0243 (11)	0.0218 (11)	0.0245 (12)	0.0052 (9)	0.0049 (9)	0.0028 (9)
C22	0.0231 (11)	0.0226 (11)	0.0227 (12)	0.0022 (9)	0.0023 (9)	0.0021 (9)
C23	0.0221 (11)	0.0265 (13)	0.0319 (14)	0.0030 (10)	0.0034 (10)	0.0004 (10)
C24	0.0271 (13)	0.0269 (13)	0.0364 (15)	0.0047 (10)	−0.0022 (11)	−0.0031 (11)
C25	0.0423 (17)	0.0458 (18)	0.0245 (14)	0.0140 (14)	−0.0001 (12)	0.0050 (12)
C26	0.0362 (15)	0.0416 (16)	0.0270 (14)	0.0135 (13)	0.0091 (12)	0.0083 (12)
Fe2	0.0267 (3)	0.0208 (2)	0.0222 (3)	0.000	0.0037 (2)	0.000
Cl2	0.0376 (4)	0.0405 (4)	0.0270 (3)	−0.0135 (3)	0.0093 (3)	−0.0080 (3)
Cl3	0.0409 (4)	0.0326 (4)	0.0387 (4)	−0.0027 (3)	0.0071 (3)	−0.0155 (3)
C27	0.026 (4)	0.030 (4)	0.101 (8)	0.000	−0.010 (4)	0.000
Cl4	0.0446 (9)	0.0845 (14)	0.0813 (14)	0.0320 (9)	0.0126 (8)	0.0329 (10)

### trans-Bis[1,2-bis(diphenylphosphane)ethylene]dichloridoiron(III) tetrachloridoferrate(III) dichloromethane 0.59-solvate (1) Geometric parameters (Å, º)

**Table:**

Fe1—Cl1^i^	2.2135 (6)	C10—C11	1.387 (4)
Fe1—Cl1	2.2135 (6)	C10—H10	0.9500
Fe1—P1	2.3662 (6)	C11—C12	1.379 (5)
Fe1—P1^i^	2.3662 (6)	C11—H11	0.9500
Fe1—P2^i^	2.3738 (6)	C12—C13	1.383 (5)
Fe1—P2	2.3738 (6)	C12—H12	0.9500
P1—C1	1.807 (3)	C13—C14	1.390 (4)
P1—C3	1.822 (6)	C13—H13	0.9500
P1—C9	1.824 (2)	C14—H14	0.9500
P1—C3'	1.832 (5)	C15—C16	1.393 (4)
P2—C2	1.811 (3)	C15—C20	1.406 (4)
P2—C21	1.825 (3)	C16—C17	1.388 (5)
P2—C15	1.829 (3)	C16—H16	0.9500
C1—C2	1.319 (4)	C17—C18	1.391 (5)
C1—H1	0.9500	C17—H17	0.9500
C2—H2	0.9500	C18—C19	1.386 (5)
C3—C8	1.379 (10)	C18—H18	0.9500
C3—C4	1.393 (10)	C19—C20	1.389 (4)
C4—C5	1.391 (10)	C19—H19	0.9500
C4—H4	0.9500	C20—H20	0.9500
C5—C6	1.369 (10)	C21—C26	1.392 (4)
C5—H5	0.9500	C21—C22	1.393 (4)
C6—C7	1.370 (10)	C22—C23	1.390 (3)
C6—H6	0.9500	C22—H22	0.9500
C7—C8	1.397 (10)	C23—C24	1.390 (4)
C7—H7	0.9500	C23—H23	0.9500
C8—H8	0.9500	C24—C25	1.383 (4)
C3'—C8'	1.396 (8)	C24—H24	0.9500
C3'—C4'	1.413 (8)	C25—C26	1.387 (4)
C4'—C5'	1.401 (8)	C25—H25	0.9500
C4'—H4'	0.9500	C26—H26	0.9500
C5'—C6'	1.368 (8)	Fe2—Cl3^ii^	2.1936 (8)
C5'—H5'	0.9500	Fe2—Cl3	2.1937 (8)
C6'—C7'	1.382 (8)	Fe2—Cl2	2.1993 (8)
C6'—H6'	0.9500	Fe2—Cl2^ii^	2.1993 (7)
C7'—C8'	1.401 (8)	C27—Cl4^iii^	1.701 (4)
C7'—H7'	0.9500	C27—Cl4	1.701 (4)
C8'—H8'	0.9500	C27—H27A	0.9900
C9—C10	1.394 (4)	C27—H27B	0.9900
C9—C14	1.395 (3)		
			
Cl1^i^—Fe1—Cl1	180.0	C3'—C8'—C7'	121.0 (6)
Cl1^i^—Fe1—P1	87.63 (2)	C3'—C8'—H8'	119.5
Cl1—Fe1—P1	92.38 (2)	C7'—C8'—H8'	119.5
Cl1^i^—Fe1—P1^i^	92.38 (2)	C10—C9—C14	118.8 (2)
Cl1—Fe1—P1^i^	87.62 (2)	C10—C9—P1	123.18 (19)
P1—Fe1—P1^i^	180.0	C14—C9—P1	118.00 (19)
Cl1^i^—Fe1—P2^i^	92.42 (2)	C11—C10—C9	120.3 (3)
Cl1—Fe1—P2^i^	87.58 (2)	C11—C10—H10	119.8
P1—Fe1—P2^i^	99.08 (2)	C9—C10—H10	119.8
P1^i^—Fe1—P2^i^	80.92 (2)	C12—C11—C10	120.1 (3)
Cl1^i^—Fe1—P2	87.58 (2)	C12—C11—H11	120.0
Cl1—Fe1—P2	92.42 (2)	C10—C11—H11	120.0
P1—Fe1—P2	80.92 (2)	C11—C12—C13	120.5 (3)
P1^i^—Fe1—P2	99.08 (2)	C11—C12—H12	119.7
P2^i^—Fe1—P2	180.0	C13—C12—H12	119.7
C1—P1—C3	97.0 (4)	C12—C13—C14	119.4 (3)
C1—P1—C9	100.81 (12)	C12—C13—H13	120.3
C3—P1—C9	108.1 (9)	C14—C13—H13	120.3
C1—P1—C3'	107.6 (3)	C13—C14—C9	120.8 (3)
C9—P1—C3'	102.4 (7)	C13—C14—H14	119.6
C1—P1—Fe1	106.98 (9)	C9—C14—H14	119.6
C3—P1—Fe1	120.7 (9)	C16—C15—C20	119.8 (3)
C9—P1—Fe1	118.90 (8)	C16—C15—P2	117.9 (2)
C3'—P1—Fe1	118.4 (7)	C20—C15—P2	122.4 (2)
C2—P2—C21	103.54 (13)	C17—C16—C15	119.8 (3)
C2—P2—C15	100.56 (14)	C17—C16—H16	120.1
C21—P2—C15	106.04 (12)	C15—C16—H16	120.1
C2—P2—Fe1	106.53 (9)	C16—C17—C18	120.5 (3)
C21—P2—Fe1	117.26 (9)	C16—C17—H17	119.7
C15—P2—Fe1	120.33 (9)	C18—C17—H17	119.7
C2—C1—P1	119.0 (2)	C19—C18—C17	119.6 (3)
C2—C1—H1	120.5	C19—C18—H18	120.2
P1—C1—H1	120.5	C17—C18—H18	120.2
C1—C2—P2	119.1 (2)	C18—C19—C20	120.6 (3)
C1—C2—H2	120.4	C18—C19—H19	119.7
P2—C2—H2	120.4	C20—C19—H19	119.7
C8—C3—C4	121.4 (7)	C19—C20—C15	119.5 (3)
C8—C3—P1	116.9 (8)	C19—C20—H20	120.3
C4—C3—P1	121.7 (8)	C15—C20—H20	120.3
C5—C4—C3	118.2 (8)	C26—C21—C22	119.0 (2)
C5—C4—H4	120.9	C26—C21—P2	121.0 (2)
C3—C4—H4	120.9	C22—C21—P2	119.92 (19)
C6—C5—C4	120.6 (8)	C23—C22—C21	120.6 (2)
C6—C5—H5	119.7	C23—C22—H22	119.7
C4—C5—H5	119.7	C21—C22—H22	119.7
C5—C6—C7	121.0 (8)	C22—C23—C24	119.8 (3)
C5—C6—H6	119.5	C22—C23—H23	120.1
C7—C6—H6	119.5	C24—C23—H23	120.1
C6—C7—C8	119.8 (8)	C25—C24—C23	119.7 (3)
C6—C7—H7	120.1	C25—C24—H24	120.1
C8—C7—H7	120.1	C23—C24—H24	120.1
C3—C8—C7	119.0 (8)	C24—C25—C26	120.5 (3)
C3—C8—H8	120.5	C24—C25—H25	119.7
C7—C8—H8	120.5	C26—C25—H25	119.7
C8'—C3'—C4'	117.9 (5)	C25—C26—C21	120.3 (3)
C8'—C3'—P1	121.9 (6)	C25—C26—H26	119.9
C4'—C3'—P1	120.2 (6)	C21—C26—H26	119.9
C5'—C4'—C3'	119.9 (6)	Cl3^ii^—Fe2—Cl3	112.36 (5)
C5'—C4'—H4'	120.0	Cl3^ii^—Fe2—Cl2	107.77 (3)
C3'—C4'—H4'	120.0	Cl3—Fe2—Cl2	110.79 (3)
C6'—C5'—C4'	121.0 (6)	Cl3^ii^—Fe2—Cl2^ii^	110.79 (3)
C6'—C5'—H5'	119.5	Cl3—Fe2—Cl2^ii^	107.76 (3)
C4'—C5'—H5'	119.5	Cl2—Fe2—Cl2^ii^	107.26 (5)
C5'—C6'—C7'	120.0 (6)	Cl4^iii^—C27—Cl4	116.2 (5)
C5'—C6'—H6'	120.0	Cl4^iii^—C27—H27A	108.2
C7'—C6'—H6'	120.0	Cl4—C27—H27A	108.2
C6'—C7'—C8'	120.0 (6)	Cl4^iii^—C27—H27B	108.2
C6'—C7'—H7'	120.0	Cl4—C27—H27B	108.2
C8'—C7'—H7'	120.0	H27A—C27—H27B	107.4
			
C3—P1—C1—C2	−105.9 (10)	C1—P1—C9—C14	−38.8 (2)
C9—P1—C1—C2	144.1 (2)	C3—P1—C9—C14	−140.0 (5)
C3'—P1—C1—C2	−109.1 (8)	C3'—P1—C9—C14	−149.7 (4)
Fe1—P1—C1—C2	19.1 (3)	Fe1—P1—C9—C14	77.6 (2)
P1—C1—C2—P2	0.6 (4)	C14—C9—C10—C11	−2.9 (4)
C21—P2—C2—C1	104.4 (3)	P1—C9—C10—C11	174.8 (2)
C15—P2—C2—C1	−146.1 (3)	C9—C10—C11—C12	1.6 (4)
Fe1—P2—C2—C1	−19.9 (3)	C10—C11—C12—C13	1.5 (5)
C1—P1—C3—C8	−177 (2)	C11—C12—C13—C14	−3.4 (4)
C9—P1—C3—C8	−74 (2)	C12—C13—C14—C9	2.1 (4)
Fe1—P1—C3—C8	68 (2)	C10—C9—C14—C13	1.0 (4)
C1—P1—C3—C4	0 (2)	P1—C9—C14—C13	−176.8 (2)
C9—P1—C3—C4	104 (2)	C2—P2—C15—C16	47.9 (3)
Fe1—P1—C3—C4	−114 (2)	C21—P2—C15—C16	155.4 (2)
C8—C3—C4—C5	0 (4)	Fe1—P2—C15—C16	−68.5 (3)
P1—C3—C4—C5	−178.0 (16)	C2—P2—C15—C20	−133.1 (3)
C3—C4—C5—C6	1 (2)	C21—P2—C15—C20	−25.5 (3)
C4—C5—C6—C7	−0.9 (19)	Fe1—P2—C15—C20	110.6 (2)
C5—C6—C7—C8	0.5 (18)	C20—C15—C16—C17	−3.8 (5)
C4—C3—C8—C7	0 (4)	P2—C15—C16—C17	175.2 (3)
P1—C3—C8—C7	177.7 (14)	C15—C16—C17—C18	0.7 (6)
C6—C7—C8—C3	0 (3)	C16—C17—C18—C19	2.8 (6)
C1—P1—C3'—C8'	−178.4 (15)	C17—C18—C19—C20	−3.1 (6)
C9—P1—C3'—C8'	−72.6 (18)	C18—C19—C20—C15	0.0 (5)
Fe1—P1—C3'—C8'	60.3 (18)	C16—C15—C20—C19	3.5 (5)
C1—P1—C3'—C4'	2.9 (18)	P2—C15—C20—C19	−175.5 (3)
C9—P1—C3'—C4'	108.6 (15)	C2—P2—C21—C26	−0.4 (3)
Fe1—P1—C3'—C4'	−118.4 (14)	C15—P2—C21—C26	−105.7 (2)
C8'—C3'—C4'—C5'	3 (2)	Fe1—P2—C21—C26	116.6 (2)
P1—C3'—C4'—C5'	−178.7 (11)	C2—P2—C21—C22	−178.3 (2)
C3'—C4'—C5'—C6'	−1.0 (18)	C15—P2—C21—C22	76.3 (2)
C4'—C5'—C6'—C7'	−0.2 (13)	Fe1—P2—C21—C22	−61.4 (2)
C5'—C6'—C7'—C8'	−0.2 (13)	C26—C21—C22—C23	−0.5 (4)
C4'—C3'—C8'—C7'	−3 (3)	P2—C21—C22—C23	177.5 (2)
P1—C3'—C8'—C7'	178.3 (11)	C21—C22—C23—C24	−1.0 (4)
C6'—C7'—C8'—C3'	1.8 (19)	C22—C23—C24—C25	1.6 (4)
C1—P1—C9—C10	143.5 (2)	C23—C24—C25—C26	−0.9 (5)
C3—P1—C9—C10	42.3 (6)	C24—C25—C26—C21	−0.6 (5)
C3'—P1—C9—C10	32.6 (5)	C22—C21—C26—C25	1.2 (4)
Fe1—P1—C9—C10	−100.1 (2)	P2—C21—C26—C25	−176.7 (2)

Symmetry codes: (i) −*x*+1, −*y*+1, −*z*+1; (ii) −*x*, *y*, −*z*+3/2; (iii) −*x*+1, *y*, −*z*+3/2.

### trans-Bis[1,2-bis(diphenylphosphane)benzene]dichloridoiron(III) tetrachloridoferrate(III) dichloromethane monosolvate (2) Crystal data

**Table:**

[FeCl_2_(C_30_H_24_P_2_)_2_][FeCl_4_]·CH_2_Cl_2_	*Z* = 1
*M**_r_* = 1302.19	*F*(000) = 664
Triclinic, *P*1	*D*_x_ = 1.473 Mg m^−^^3^
*a* = 9.8771 (7) Å	Mo *K*α radiation, λ = 0.71073 Å
*b* = 12.6516 (8) Å	Cell parameters from 4051 reflections
*c* = 12.8258 (8) Å	θ = 2.3–30.7°
α = 81.058 (1)°	µ = 1.01 mm^−^^1^
β = 83.050 (1)°	*T* = 100 K
γ = 68.335 (1)°	Plate, dichroic red-green
*V* = 1467.87 (17) Å^3^	0.24 × 0.20 × 0.08 mm

### trans-Bis[1,2-bis(diphenylphosphane)benzene]dichloridoiron(III) tetrachloridoferrate(III) dichloromethane monosolvate (2) Data collection

**Table:**

Bruker SMART APEX II CCD platform diffractometer	11890 reflections with *I* > 2σ(*I*)
Radiation source: fine-focus sealed tube	*R*_int_ = 0.050
ω scans	θ_max_ = 35.0°, θ_min_ = 2.2°
Absorption correction: multi-scan (SADABS; Krause *et al*., 2015)	*h* = −15→15
*T*_min_ = 0.644, *T*_max_ = 0.747	*k* = −20→20
27211 measured reflections	*l* = −20→18
22268 independent reflections	

### trans-Bis[1,2-bis(diphenylphosphane)benzene]dichloridoiron(III) tetrachloridoferrate(III) dichloromethane monosolvate (2) Refinement

**Table:**

Refinement on *F*^2^	Secondary atom site location: difference Fourier map
Least-squares matrix: full	Hydrogen site location: inferred from neighbouring sites
*R*[*F*^2^ > 2σ(*F*^2^)] = 0.073	H-atom parameters constrained
*wR*(*F*^2^) = 0.182	*w* = 1/[σ^2^(*F*_o_^2^) + (0.0641*P*)^2^] where *P* = (*F*_o_^2^ + 2*F*_c_^2^)/3
*S* = 1.00	(Δ/σ)_max_ < 0.001
22268 reflections	Δρ_max_ = 1.23 e Å^−^^3^
488 parameters	Δρ_min_ = −1.39 e Å^−^^3^
34 restraints	Absolute structure: Refined as an inversion twin.
Primary atom site location: dual	Absolute structure parameter: 0.24 (3)

### trans-Bis[1,2-bis(diphenylphosphane)benzene]dichloridoiron(III) tetrachloridoferrate(III) dichloromethane monosolvate (2) Special details

**Table:**

Geometry. All esds (except the esd in the dihedral angle between two l.s. planes) are estimated using the full covariance matrix. The cell esds are taken into account individually in the estimation of esds in distances, angles and torsion angles; correlations between esds in cell parameters are only used when they are defined by crystal symmetry. An approximate (isotropic) treatment of cell esds is used for estimating esds involving l.s. planes.
Refinement. The structure was modeled as an inversion twin whose component mass ratio refined to 0.76 (3):0.24 (3). A solution and refinement in centrosymmetric space group P-1 caused an increase in the R1 residual (strong data) from 0.071 to 0.118. The cocrystallized dichloromethane solvent molecule is modeled as disordered over three positions (0.740 (3):0.136 (3):0.124 (3)). Analogous bond lengths and angles among the three positions of the disordered dichloromethane solvent molecule were restrained to be similar. Anisotropic displacement parameters for proximal and pseudosymmetrically related atoms were constrained to be equivalent.

### trans-Bis[1,2-bis(diphenylphosphane)benzene]dichloridoiron(III) tetrachloridoferrate(III) dichloromethane monosolvate (2) Fractional atomic coordinates and isotropic or equivalent isotropic displacement parameters (Å^2^)

**Table:**

	*x*	*y*	*z*	*U*_iso_*/*U*_eq_	Occ. (<1)
Fe1	0.50286 (14)	0.39706 (10)	0.63842 (10)	0.01341 (15)	
Cl1	0.68914 (19)	0.42220 (14)	0.69888 (14)	0.01712 (17)	
Cl2	0.31700 (19)	0.37173 (15)	0.57842 (14)	0.01712 (17)	
P1	0.3591 (2)	0.59048 (15)	0.66544 (16)	0.01471 (18)	
P2	0.5609 (2)	0.48921 (15)	0.47244 (15)	0.01494 (19)	
P3	0.6449 (2)	0.20476 (15)	0.61113 (16)	0.01471 (18)	
P4	0.4468 (2)	0.30281 (15)	0.80391 (15)	0.01494 (19)	
C1	0.4350 (8)	0.6828 (6)	0.5755 (6)	0.0168 (7)	
C2	0.4012 (9)	0.7982 (6)	0.5880 (7)	0.0203 (8)	
H2	0.342059	0.829432	0.647917	0.024*	
C3	0.4557 (9)	0.8671 (7)	0.5111 (7)	0.0224 (8)	
H3	0.430915	0.945688	0.518605	0.027*	
C4	0.5440 (10)	0.8228 (7)	0.4253 (7)	0.0230 (8)	
H4	0.580837	0.870627	0.374680	0.028*	
C5	0.5801 (10)	0.7077 (7)	0.4120 (7)	0.0231 (8)	
H5	0.641033	0.677084	0.352542	0.028*	
C6	0.5258 (9)	0.6381 (6)	0.4871 (6)	0.0168 (7)	
C7	0.3577 (9)	0.6416 (6)	0.7913 (6)	0.0170 (7)	
C8	0.4891 (9)	0.6487 (6)	0.8184 (7)	0.0185 (7)	
H8	0.573481	0.629636	0.770516	0.022*	
C9	0.4942 (10)	0.6834 (7)	0.9141 (7)	0.0216 (8)	
H9	0.582948	0.686509	0.932254	0.026*	
C10	0.3734 (10)	0.7134 (7)	0.9833 (7)	0.0253 (9)	
H10	0.378826	0.736978	1.049025	0.030*	
C11	0.2439 (10)	0.7094 (7)	0.9579 (7)	0.0276 (10)	
H11	0.159982	0.730938	1.005926	0.033*	
C12	0.2351 (9)	0.6736 (7)	0.8613 (7)	0.0217 (8)	
H12	0.145437	0.671463	0.843936	0.026*	
C13	0.1675 (9)	0.6443 (7)	0.6345 (7)	0.0205 (8)	
C14	0.0746 (9)	0.5937 (7)	0.6896 (7)	0.0234 (8)	
H14	0.110682	0.531331	0.743055	0.028*	
C15	−0.0721 (10)	0.6326 (8)	0.6681 (8)	0.0292 (10)	
H15	−0.134976	0.597322	0.707755	0.035*	
C16	−0.1257 (10)	0.7200 (8)	0.5913 (8)	0.0308 (10)	
H16	−0.225523	0.745221	0.576744	0.037*	
C17	−0.0333 (11)	0.7737 (8)	0.5330 (8)	0.0358 (11)	
H17	−0.069872	0.834910	0.478609	0.043*	
C18	0.1131 (10)	0.7356 (7)	0.5564 (7)	0.0272 (9)	
H18	0.175868	0.772242	0.518776	0.033*	
C19	0.4506 (8)	0.5019 (6)	0.3642 (6)	0.0165 (7)	
C20	0.3450 (9)	0.6064 (7)	0.3297 (7)	0.0254 (9)	
H20	0.333632	0.672703	0.361033	0.030*	
C21	0.2576 (10)	0.6151 (8)	0.2516 (7)	0.0292 (10)	
H21	0.184693	0.686828	0.230899	0.035*	
C22	0.2737 (10)	0.5204 (8)	0.2020 (7)	0.0262 (10)	
H22	0.215100	0.527670	0.145873	0.031*	
C23	0.3766 (9)	0.4155 (7)	0.2358 (6)	0.0206 (9)	
H23	0.389282	0.349815	0.202910	0.025*	
C24	0.4610 (9)	0.4065 (7)	0.3178 (6)	0.0187 (8)	
H24	0.527743	0.333368	0.342963	0.022*	
C25	0.7486 (9)	0.4461 (7)	0.4165 (6)	0.0177 (7)	
C26	0.7991 (9)	0.3814 (7)	0.3313 (7)	0.0246 (9)	
H26	0.732100	0.362996	0.296982	0.030*	
C27	0.9467 (10)	0.3436 (9)	0.2961 (8)	0.0345 (11)	
H27	0.981402	0.297850	0.239587	0.041*	
C28	1.0437 (10)	0.3753 (9)	0.3469 (8)	0.0346 (12)	
H28	1.144813	0.348675	0.324980	0.041*	
C29	0.9948 (10)	0.4427 (8)	0.4259 (8)	0.0303 (11)	
H29	1.060584	0.465935	0.456527	0.036*	
C30	0.8479 (9)	0.4781 (7)	0.4623 (7)	0.0228 (8)	
H30	0.814661	0.524277	0.518586	0.027*	
C31	0.5655 (8)	0.1115 (6)	0.7002 (6)	0.0168 (7)	
C32	0.6008 (9)	−0.0039 (7)	0.6850 (7)	0.0203 (8)	
H32	0.661938	−0.033872	0.625422	0.024*	
C33	0.5457 (9)	−0.0727 (7)	0.7576 (7)	0.0224 (8)	
H33	0.568623	−0.150634	0.748200	0.027*	
C34	0.4560 (10)	−0.0278 (7)	0.8449 (7)	0.0230 (8)	
H34	0.417449	−0.075317	0.894485	0.028*	
C35	0.4230 (10)	0.0844 (7)	0.8600 (7)	0.0231 (8)	
H35	0.363418	0.113538	0.920434	0.028*	
C36	0.4772 (9)	0.1564 (6)	0.7861 (6)	0.0168 (7)	
C37	0.6454 (9)	0.1559 (6)	0.4842 (6)	0.0170 (7)	
C38	0.5193 (9)	0.1451 (6)	0.4567 (7)	0.0185 (7)	
H38	0.435397	0.161863	0.505053	0.022*	
C39	0.5133 (10)	0.1107 (7)	0.3611 (7)	0.0216 (8)	
H39	0.426161	0.104035	0.343895	0.026*	
C40	0.6362 (10)	0.0858 (7)	0.2894 (7)	0.0253 (9)	
H40	0.632232	0.063705	0.222680	0.030*	
C41	0.7640 (10)	0.0934 (7)	0.3162 (7)	0.0276 (10)	
H41	0.848092	0.075253	0.268098	0.033*	
C42	0.7695 (9)	0.1273 (7)	0.4130 (7)	0.0217 (8)	
H42	0.857814	0.131138	0.431233	0.026*	
C43	0.8368 (9)	0.1487 (7)	0.6415 (7)	0.0205 (8)	
C44	0.9319 (9)	0.2031 (7)	0.5899 (7)	0.0234 (8)	
H44	0.894856	0.268358	0.539493	0.028*	
C45	1.0755 (10)	0.1651 (8)	0.6101 (8)	0.0292 (10)	
H45	1.138383	0.201914	0.572957	0.035*	
C46	1.1292 (10)	0.0697 (8)	0.6873 (8)	0.0308 (10)	
H46	1.228576	0.042808	0.703517	0.037*	
C47	1.0388 (11)	0.0167 (8)	0.7381 (8)	0.0358 (11)	
H47	1.076749	−0.048303	0.788638	0.043*	
C48	0.8941 (10)	0.0544 (7)	0.7185 (7)	0.0272 (9)	
H48	0.832507	0.016987	0.756782	0.033*	
C49	0.5653 (8)	0.2851 (6)	0.9099 (6)	0.0165 (7)	
C50	0.6609 (10)	0.1782 (7)	0.9469 (7)	0.0254 (9)	
H50	0.664284	0.112121	0.918717	0.030*	
C51	0.7527 (10)	0.1675 (8)	1.0259 (7)	0.0292 (10)	
H51	0.819167	0.094181	1.050727	0.035*	
C52	0.7467 (10)	0.2646 (8)	1.0683 (7)	0.0262 (10)	
H52	0.809821	0.257491	1.121332	0.031*	
C53	0.6490 (9)	0.3706 (7)	1.0329 (6)	0.0206 (9)	
H53	0.642575	0.436063	1.063628	0.025*	
C54	0.5595 (9)	0.3826 (7)	0.9523 (6)	0.0187 (8)	
H54	0.495090	0.456324	0.926328	0.022*	
C55	0.2583 (9)	0.3503 (7)	0.8622 (6)	0.0177 (7)	
C56	0.2117 (9)	0.4183 (7)	0.9441 (7)	0.0246 (9)	
H56	0.280279	0.438387	0.974875	0.030*	
C57	0.0671 (10)	0.4570 (9)	0.9813 (8)	0.0345 (11)	
H57	0.037337	0.503542	1.037323	0.041*	
C58	−0.0343 (10)	0.4296 (8)	0.9388 (8)	0.0346 (12)	
H58	−0.134230	0.458287	0.963586	0.041*	
C59	0.0112 (10)	0.3588 (8)	0.8584 (8)	0.0303 (11)	
H59	−0.057673	0.337013	0.829994	0.036*	
C60	0.1560 (9)	0.3200 (7)	0.8196 (7)	0.0228 (8)	
H60	0.185630	0.272858	0.764084	0.027*	
Fe2	0.74909 (13)	0.79207 (11)	0.09572 (11)	0.0302 (3)	
Cl3	0.5310 (2)	0.92270 (18)	0.11580 (17)	0.0339 (4)	
Cl4	0.7333 (3)	0.62264 (18)	0.13622 (18)	0.0437 (6)	
Cl5	0.8376 (3)	0.8101 (3)	−0.0681 (3)	0.0645 (6)	
Cl6	0.8890 (4)	0.8166 (3)	0.2034 (4)	0.0963 (15)	
Cl7	0.1337 (4)	0.0699 (5)	0.1131 (4)	0.0819 (15)	0.740 (3)
C61	0.1765 (12)	−0.0475 (9)	0.2177 (8)	0.0302 (3)	0.740 (3)
H61A	0.108903	−0.089082	0.218036	0.036*	0.740 (3)
H61B	0.276777	−0.101351	0.202280	0.036*	0.740 (3)
Cl8	0.1656 (4)	−0.0086 (4)	0.3389 (4)	0.0645 (6)	0.740 (3)
Cl7'	0.129 (3)	−0.012 (3)	0.087 (3)	0.0963 (15)	0.124 (3)
C61'	0.249 (5)	0.007 (7)	0.168 (3)	0.0302 (3)	0.124 (3)
H61C	0.332305	−0.066406	0.178368	0.036*	0.124 (3)
H61D	0.287840	0.064322	0.128480	0.036*	0.124 (3)
Cl8'	0.178 (2)	0.050 (2)	0.2923 (19)	0.0645 (6)	0.124 (3)
Cl7"	0.127 (2)	−0.0679 (19)	0.166 (2)	0.0645 (6)	0.136 (3)
C61"	0.276 (4)	−0.026 (3)	0.178 (6)	0.0302 (3)	0.136 (3)
H61E	0.327391	−0.066354	0.242501	0.036*	0.136 (3)
H61F	0.345977	−0.040293	0.115397	0.036*	0.136 (3)
Cl8"	0.191 (2)	0.119 (2)	0.187 (2)	0.0819 (15)	0.136 (3)

### trans-Bis[1,2-bis(diphenylphosphane)benzene]dichloridoiron(III) tetrachloridoferrate(III) dichloromethane monosolvate (2) Atomic displacement parameters (Å^2^)

**Table:**

	*U*^11^	*U*^22^	*U*^33^	*U*^12^	*U*^13^	*U*^23^
Fe1	0.0152 (4)	0.0115 (3)	0.0147 (4)	−0.0053 (3)	−0.0038 (3)	−0.0015 (3)
Cl1	0.0180 (4)	0.0166 (4)	0.0190 (4)	−0.0078 (3)	−0.0047 (3)	−0.0020 (3)
Cl2	0.0180 (4)	0.0166 (4)	0.0190 (4)	−0.0078 (3)	−0.0047 (3)	−0.0020 (3)
P1	0.0166 (4)	0.0123 (4)	0.0160 (5)	−0.0052 (3)	−0.0034 (4)	−0.0018 (3)
P2	0.0181 (5)	0.0128 (4)	0.0151 (5)	−0.0064 (4)	−0.0035 (4)	−0.0012 (3)
P3	0.0166 (4)	0.0123 (4)	0.0160 (5)	−0.0052 (3)	−0.0034 (4)	−0.0018 (3)
P4	0.0181 (5)	0.0128 (4)	0.0151 (5)	−0.0064 (4)	−0.0035 (4)	−0.0012 (3)
C1	0.0207 (18)	0.0144 (15)	0.0161 (18)	−0.0071 (14)	−0.0032 (14)	−0.0003 (13)
C2	0.027 (2)	0.0151 (16)	0.021 (2)	−0.0076 (15)	−0.0045 (16)	−0.0037 (14)
C3	0.030 (2)	0.0132 (16)	0.024 (2)	−0.0072 (15)	−0.0075 (17)	−0.0012 (15)
C4	0.032 (2)	0.0181 (17)	0.022 (2)	−0.0130 (17)	−0.0054 (17)	0.0039 (15)
C5	0.032 (2)	0.0185 (18)	0.020 (2)	−0.0115 (17)	−0.0020 (17)	−0.0025 (15)
C6	0.0188 (18)	0.0146 (15)	0.0169 (18)	−0.0053 (14)	−0.0030 (14)	−0.0020 (13)
C7	0.0207 (18)	0.0116 (15)	0.0191 (19)	−0.0052 (13)	−0.0045 (14)	−0.0017 (13)
C8	0.0198 (18)	0.0149 (16)	0.023 (2)	−0.0078 (14)	−0.0034 (15)	−0.0035 (14)
C9	0.031 (2)	0.0160 (16)	0.021 (2)	−0.0115 (16)	−0.0094 (16)	0.0006 (14)
C10	0.040 (3)	0.0204 (19)	0.019 (2)	−0.0132 (18)	−0.0048 (18)	−0.0042 (15)
C11	0.033 (2)	0.029 (2)	0.023 (2)	−0.0135 (19)	0.0038 (18)	−0.0086 (18)
C12	0.022 (2)	0.0203 (19)	0.023 (2)	−0.0073 (16)	−0.0021 (16)	−0.0038 (15)
C13	0.0206 (19)	0.0171 (17)	0.024 (2)	−0.0051 (15)	−0.0047 (15)	−0.0046 (14)
C14	0.022 (2)	0.0182 (18)	0.033 (2)	−0.0090 (15)	−0.0062 (17)	−0.0031 (16)
C15	0.020 (2)	0.029 (2)	0.042 (3)	−0.0091 (18)	−0.0049 (19)	−0.012 (2)
C16	0.019 (2)	0.029 (2)	0.042 (3)	−0.0007 (17)	−0.0151 (19)	−0.008 (2)
C17	0.030 (3)	0.029 (2)	0.040 (3)	0.0013 (19)	−0.019 (2)	0.003 (2)
C18	0.026 (2)	0.023 (2)	0.028 (2)	−0.0032 (17)	−0.0068 (18)	0.0002 (17)
C19	0.0190 (19)	0.0193 (17)	0.0110 (17)	−0.0068 (15)	−0.0015 (14)	−0.0008 (13)
C20	0.029 (2)	0.020 (2)	0.025 (2)	−0.0047 (17)	−0.0074 (17)	−0.0054 (17)
C21	0.027 (2)	0.026 (2)	0.031 (3)	−0.0029 (19)	−0.0128 (19)	−0.0014 (19)
C22	0.029 (2)	0.035 (2)	0.017 (2)	−0.014 (2)	−0.0094 (18)	0.0011 (17)
C23	0.024 (2)	0.026 (2)	0.017 (2)	−0.0141 (19)	0.0009 (17)	−0.0056 (16)
C24	0.019 (2)	0.0191 (17)	0.020 (2)	−0.0105 (16)	−0.0017 (16)	0.0001 (14)
C25	0.0182 (18)	0.0185 (17)	0.0173 (19)	−0.0081 (14)	−0.0023 (14)	−0.0002 (13)
C26	0.022 (2)	0.027 (2)	0.027 (2)	−0.0099 (17)	−0.0006 (17)	−0.0070 (17)
C27	0.026 (2)	0.040 (3)	0.038 (3)	−0.012 (2)	0.006 (2)	−0.013 (2)
C28	0.023 (2)	0.043 (3)	0.041 (3)	−0.017 (2)	0.005 (2)	−0.007 (2)
C29	0.024 (2)	0.034 (2)	0.038 (3)	−0.019 (2)	−0.007 (2)	0.002 (2)
C30	0.028 (2)	0.026 (2)	0.021 (2)	−0.0169 (17)	−0.0042 (16)	0.0003 (16)
C31	0.0207 (18)	0.0144 (15)	0.0161 (18)	−0.0071 (14)	−0.0032 (14)	−0.0003 (13)
C32	0.027 (2)	0.0151 (16)	0.021 (2)	−0.0076 (15)	−0.0045 (16)	−0.0037 (14)
C33	0.030 (2)	0.0132 (16)	0.024 (2)	−0.0072 (15)	−0.0075 (17)	−0.0012 (15)
C34	0.032 (2)	0.0181 (17)	0.022 (2)	−0.0130 (17)	−0.0054 (17)	0.0039 (15)
C35	0.032 (2)	0.0185 (18)	0.020 (2)	−0.0115 (17)	−0.0020 (17)	−0.0025 (15)
C36	0.0188 (18)	0.0146 (15)	0.0169 (18)	−0.0053 (14)	−0.0030 (14)	−0.0020 (13)
C37	0.0207 (18)	0.0116 (15)	0.0191 (19)	−0.0052 (13)	−0.0045 (14)	−0.0017 (13)
C38	0.0198 (18)	0.0149 (16)	0.023 (2)	−0.0078 (14)	−0.0034 (15)	−0.0035 (14)
C39	0.031 (2)	0.0160 (16)	0.021 (2)	−0.0115 (16)	−0.0094 (16)	0.0006 (14)
C40	0.040 (3)	0.0204 (19)	0.019 (2)	−0.0132 (18)	−0.0048 (18)	−0.0042 (15)
C41	0.033 (2)	0.029 (2)	0.023 (2)	−0.0135 (19)	0.0038 (18)	−0.0086 (18)
C42	0.022 (2)	0.0203 (19)	0.023 (2)	−0.0073 (16)	−0.0021 (16)	−0.0038 (15)
C43	0.0206 (19)	0.0171 (17)	0.024 (2)	−0.0051 (15)	−0.0047 (15)	−0.0046 (14)
C44	0.022 (2)	0.0182 (18)	0.033 (2)	−0.0090 (15)	−0.0062 (17)	−0.0031 (16)
C45	0.020 (2)	0.029 (2)	0.042 (3)	−0.0091 (18)	−0.0049 (19)	−0.012 (2)
C46	0.019 (2)	0.029 (2)	0.042 (3)	−0.0007 (17)	−0.0151 (19)	−0.008 (2)
C47	0.030 (3)	0.029 (2)	0.040 (3)	0.0013 (19)	−0.019 (2)	0.003 (2)
C48	0.026 (2)	0.023 (2)	0.028 (2)	−0.0032 (17)	−0.0068 (18)	0.0002 (17)
C49	0.0190 (19)	0.0193 (17)	0.0110 (17)	−0.0068 (15)	−0.0015 (14)	−0.0008 (13)
C50	0.029 (2)	0.020 (2)	0.025 (2)	−0.0047 (17)	−0.0074 (17)	−0.0054 (17)
C51	0.027 (2)	0.026 (2)	0.031 (3)	−0.0029 (19)	−0.0128 (19)	−0.0014 (19)
C52	0.029 (2)	0.035 (2)	0.017 (2)	−0.014 (2)	−0.0094 (18)	0.0011 (17)
C53	0.024 (2)	0.026 (2)	0.017 (2)	−0.0141 (19)	0.0009 (17)	−0.0056 (16)
C54	0.019 (2)	0.0191 (17)	0.020 (2)	−0.0105 (16)	−0.0017 (16)	0.0001 (14)
C55	0.0182 (18)	0.0185 (17)	0.0173 (19)	−0.0081 (14)	−0.0023 (14)	−0.0002 (13)
C56	0.022 (2)	0.027 (2)	0.027 (2)	−0.0099 (17)	−0.0006 (17)	−0.0070 (17)
C57	0.026 (2)	0.040 (3)	0.038 (3)	−0.012 (2)	0.006 (2)	−0.013 (2)
C58	0.023 (2)	0.043 (3)	0.041 (3)	−0.017 (2)	0.005 (2)	−0.007 (2)
C59	0.024 (2)	0.034 (2)	0.038 (3)	−0.019 (2)	−0.007 (2)	0.002 (2)
C60	0.028 (2)	0.026 (2)	0.021 (2)	−0.0169 (17)	−0.0042 (16)	0.0003 (16)
Fe2	0.0234 (6)	0.0251 (6)	0.0393 (7)	−0.0018 (4)	−0.0095 (5)	−0.0077 (5)
Cl3	0.0226 (9)	0.0378 (11)	0.0356 (12)	−0.0001 (8)	−0.0087 (8)	−0.0093 (9)
Cl4	0.0709 (17)	0.0271 (10)	0.0283 (11)	−0.0138 (11)	0.0042 (11)	−0.0046 (8)
Cl5	0.0335 (9)	0.0725 (13)	0.0668 (13)	−0.0075 (9)	0.0006 (8)	0.0200 (10)
Cl6	0.0488 (16)	0.090 (2)	0.146 (3)	0.0219 (15)	−0.0621 (19)	−0.077 (2)
Cl7	0.0330 (19)	0.128 (4)	0.070 (3)	−0.006 (2)	−0.0002 (18)	−0.027 (3)
C61	0.0234 (6)	0.0251 (6)	0.0393 (7)	−0.0018 (4)	−0.0095 (5)	−0.0077 (5)
Cl8	0.0335 (9)	0.0725 (13)	0.0668 (13)	−0.0075 (9)	0.0006 (8)	0.0200 (10)
Cl7'	0.0488 (16)	0.090 (2)	0.146 (3)	0.0219 (15)	−0.0621 (19)	−0.077 (2)
C61'	0.0234 (6)	0.0251 (6)	0.0393 (7)	−0.0018 (4)	−0.0095 (5)	−0.0077 (5)
Cl8'	0.0335 (9)	0.0725 (13)	0.0668 (13)	−0.0075 (9)	0.0006 (8)	0.0200 (10)
Cl7"	0.0335 (9)	0.0725 (13)	0.0668 (13)	−0.0075 (9)	0.0006 (8)	0.0200 (10)
C61"	0.0234 (6)	0.0251 (6)	0.0393 (7)	−0.0018 (4)	−0.0095 (5)	−0.0077 (5)
Cl8"	0.0330 (19)	0.128 (4)	0.070 (3)	−0.006 (2)	−0.0002 (18)	−0.027 (3)

### trans-Bis[1,2-bis(diphenylphosphane)benzene]dichloridoiron(III) tetrachloridoferrate(III) dichloromethane monosolvate (2) Geometric parameters (Å, º)

**Table:**

Fe1—Cl2	2.218 (2)	C30—H30	0.9500
Fe1—Cl1	2.223 (2)	C31—C36	1.376 (11)
Fe1—P3	2.374 (2)	C31—C32	1.410 (10)
Fe1—P2	2.376 (2)	C32—C33	1.377 (11)
Fe1—P4	2.377 (2)	C32—H32	0.9500
Fe1—P1	2.388 (2)	C33—C34	1.396 (12)
P1—C1	1.810 (8)	C33—H33	0.9500
P1—C7	1.827 (8)	C34—C35	1.374 (11)
P1—C13	1.830 (8)	C34—H34	0.9500
P2—C25	1.817 (8)	C35—C36	1.408 (11)
P2—C19	1.819 (8)	C35—H35	0.9500
P2—C6	1.822 (7)	C37—C38	1.393 (11)
P3—C37	1.828 (8)	C37—C42	1.404 (11)
P3—C43	1.830 (8)	C38—C39	1.379 (11)
P3—C31	1.833 (8)	C38—H38	0.9500
P4—C36	1.811 (7)	C39—C40	1.398 (13)
P4—C49	1.834 (8)	C39—H39	0.9500
P4—C55	1.835 (8)	C40—C41	1.387 (12)
C1—C2	1.403 (10)	C40—H40	0.9500
C1—C6	1.410 (11)	C41—C42	1.389 (11)
C2—C3	1.404 (11)	C41—H41	0.9500
C2—H2	0.9500	C42—H42	0.9500
C3—C4	1.373 (12)	C43—C44	1.406 (11)
C3—H3	0.9500	C43—C48	1.411 (12)
C4—C5	1.399 (11)	C44—C45	1.362 (11)
C4—H4	0.9500	C44—H44	0.9500
C5—C6	1.396 (11)	C45—C46	1.415 (13)
C5—H5	0.9500	C45—H45	0.9500
C7—C12	1.387 (11)	C46—C47	1.355 (13)
C7—C8	1.421 (11)	C46—H46	0.9500
C8—C9	1.379 (10)	C47—C48	1.371 (12)
C8—H8	0.9500	C47—H47	0.9500
C9—C10	1.367 (12)	C48—H48	0.9500
C9—H9	0.9500	C49—C50	1.382 (11)
C10—C11	1.379 (12)	C49—C54	1.404 (10)
C10—H10	0.9500	C50—C51	1.398 (12)
C11—C12	1.406 (11)	C50—H50	0.9500
C11—H11	0.9500	C51—C52	1.398 (11)
C12—H12	0.9500	C51—H51	0.9500
C13—C14	1.374 (11)	C52—C53	1.377 (12)
C13—C18	1.392 (12)	C52—H52	0.9500
C14—C15	1.394 (11)	C53—C54	1.395 (11)
C14—H14	0.9500	C53—H53	0.9500
C15—C16	1.352 (13)	C54—H54	0.9500
C15—H15	0.9500	C55—C56	1.388 (11)
C16—C17	1.411 (14)	C55—C60	1.396 (11)
C16—H16	0.9500	C56—C57	1.378 (12)
C17—C18	1.400 (12)	C56—H56	0.9500
C17—H17	0.9500	C57—C58	1.367 (12)
C18—H18	0.9500	C57—H57	0.9500
C19—C24	1.392 (10)	C58—C59	1.394 (13)
C19—C20	1.394 (11)	C58—H58	0.9500
C20—C21	1.365 (12)	C59—C60	1.385 (12)
C20—H20	0.9500	C59—H59	0.9500
C21—C22	1.392 (12)	C60—H60	0.9500
C21—H21	0.9500	Fe2—Cl4	2.184 (2)
C22—C23	1.384 (12)	Fe2—Cl5	2.188 (3)
C22—H22	0.9500	Fe2—Cl3	2.193 (2)
C23—C24	1.385 (11)	Fe2—Cl6	2.197 (3)
C23—H23	0.9500	Cl7—C61	1.799 (11)
C24—H24	0.9500	C61—Cl8	1.682 (10)
C25—C26	1.399 (11)	C61—H61A	0.9900
C25—C30	1.406 (11)	C61—H61B	0.9900
C26—C27	1.397 (12)	Cl7'—C61'	1.77 (2)
C26—H26	0.9500	C61'—Cl8'	1.75 (2)
C27—C28	1.420 (13)	C61'—H61C	0.9900
C27—H27	0.9500	C61'—H61D	0.9900
C28—C29	1.354 (13)	Cl7"—C61"	1.76 (2)
C28—H28	0.9500	C61"—Cl8"	1.73 (2)
C29—C30	1.394 (12)	C61"—H61E	0.9900
C29—H29	0.9500	C61"—H61F	0.9900
			
Cl2—Fe1—Cl1	179.87 (12)	C27—C28—H28	119.4
Cl2—Fe1—P3	87.69 (8)	C28—C29—C30	120.1 (8)
Cl1—Fe1—P3	92.26 (8)	C28—C29—H29	119.9
Cl2—Fe1—P2	92.82 (8)	C30—C29—H29	119.9
Cl1—Fe1—P2	87.30 (8)	C29—C30—C25	120.7 (8)
P3—Fe1—P2	98.58 (8)	C29—C30—H30	119.7
Cl2—Fe1—P4	87.23 (8)	C25—C30—H30	119.7
Cl1—Fe1—P4	92.65 (8)	C36—C31—C32	121.2 (7)
P3—Fe1—P4	80.75 (8)	C36—C31—P3	117.5 (6)
P2—Fe1—P4	179.33 (10)	C32—C31—P3	121.2 (6)
Cl2—Fe1—P1	92.05 (8)	C33—C32—C31	119.3 (7)
Cl1—Fe1—P1	87.99 (7)	C33—C32—H32	120.4
P3—Fe1—P1	179.74 (10)	C31—C32—H32	120.4
P2—Fe1—P1	81.38 (8)	C32—C33—C34	119.9 (7)
P4—Fe1—P1	99.29 (8)	C32—C33—H33	120.1
C1—P1—C7	100.8 (3)	C34—C33—H33	120.1
C1—P1—C13	103.2 (4)	C35—C34—C33	120.7 (8)
C7—P1—C13	105.0 (4)	C35—C34—H34	119.7
C1—P1—Fe1	107.7 (3)	C33—C34—H34	119.7
C7—P1—Fe1	120.0 (3)	C34—C35—C36	120.3 (8)
C13—P1—Fe1	117.6 (2)	C34—C35—H35	119.9
C25—P2—C19	105.8 (4)	C36—C35—H35	119.9
C25—P2—C6	100.4 (4)	C31—C36—C35	118.7 (7)
C19—P2—C6	102.7 (4)	C31—C36—P4	118.2 (6)
C25—P2—Fe1	120.0 (3)	C35—C36—P4	122.9 (6)
C19—P2—Fe1	117.0 (3)	C38—C37—C42	118.3 (7)
C6—P2—Fe1	108.3 (3)	C38—C37—P3	119.4 (6)
C37—P3—C43	104.8 (4)	C42—C37—P3	122.3 (6)
C37—P3—C31	100.6 (3)	C39—C38—C37	121.6 (8)
C43—P3—C31	103.8 (4)	C39—C38—H38	119.2
C37—P3—Fe1	119.4 (3)	C37—C38—H38	119.2
C43—P3—Fe1	118.2 (2)	C38—C39—C40	119.7 (8)
C31—P3—Fe1	107.7 (3)	C38—C39—H39	120.2
C36—P4—C49	102.8 (4)	C40—C39—H39	120.2
C36—P4—C55	101.4 (4)	C41—C40—C39	119.7 (8)
C49—P4—C55	107.0 (4)	C41—C40—H40	120.1
C36—P4—Fe1	108.0 (3)	C39—C40—H40	120.1
C49—P4—Fe1	116.1 (3)	C40—C41—C42	120.3 (8)
C55—P4—Fe1	119.2 (3)	C40—C41—H41	119.9
C2—C1—C6	119.1 (7)	C42—C41—H41	119.9
C2—C1—P1	122.3 (6)	C41—C42—C37	120.5 (8)
C6—C1—P1	118.5 (6)	C41—C42—H42	119.8
C1—C2—C3	119.3 (7)	C37—C42—H42	119.8
C1—C2—H2	120.3	C44—C43—C48	117.6 (8)
C3—C2—H2	120.3	C44—C43—P3	119.8 (6)
C4—C3—C2	121.2 (7)	C48—C43—P3	122.6 (7)
C4—C3—H3	119.4	C45—C44—C43	122.0 (9)
C2—C3—H3	119.4	C45—C44—H44	119.0
C3—C4—C5	120.3 (8)	C43—C44—H44	119.0
C3—C4—H4	119.8	C44—C45—C46	118.7 (9)
C5—C4—H4	119.8	C44—C45—H45	120.6
C6—C5—C4	119.3 (8)	C46—C45—H45	120.6
C6—C5—H5	120.3	C47—C46—C45	119.9 (8)
C4—C5—H5	120.3	C47—C46—H46	120.0
C5—C6—C1	120.7 (7)	C45—C46—H46	120.0
C5—C6—P2	122.0 (6)	C46—C47—C48	121.8 (9)
C1—C6—P2	117.2 (6)	C46—C47—H47	119.1
C12—C7—C8	118.7 (7)	C48—C47—H47	119.1
C12—C7—P1	123.3 (6)	C47—C48—C43	119.9 (9)
C8—C7—P1	118.1 (6)	C47—C48—H48	120.1
C9—C8—C7	119.9 (8)	C43—C48—H48	120.1
C9—C8—H8	120.0	C50—C49—C54	119.9 (7)
C7—C8—H8	120.0	C50—C49—P4	121.2 (6)
C10—C9—C8	121.1 (8)	C54—C49—P4	118.9 (6)
C10—C9—H9	119.5	C49—C50—C51	120.0 (7)
C8—C9—H9	119.5	C49—C50—H50	120.0
C9—C10—C11	120.0 (8)	C51—C50—H50	120.0
C9—C10—H10	120.0	C50—C51—C52	120.0 (8)
C11—C10—H10	120.0	C50—C51—H51	120.0
C10—C11—C12	120.4 (8)	C52—C51—H51	120.0
C10—C11—H11	119.8	C53—C52—C51	119.8 (8)
C12—C11—H11	119.8	C53—C52—H52	120.1
C7—C12—C11	119.9 (8)	C51—C52—H52	120.1
C7—C12—H12	120.1	C52—C53—C54	120.6 (7)
C11—C12—H12	120.1	C52—C53—H53	119.7
C14—C13—C18	119.0 (8)	C54—C53—H53	119.7
C14—C13—P1	119.4 (7)	C53—C54—C49	119.6 (8)
C18—C13—P1	121.6 (7)	C53—C54—H54	120.2
C13—C14—C15	120.8 (9)	C49—C54—H54	120.2
C13—C14—H14	119.6	C56—C55—C60	118.6 (8)
C15—C14—H14	119.6	C56—C55—P4	122.9 (6)
C16—C15—C14	120.8 (9)	C60—C55—P4	118.4 (6)
C16—C15—H15	119.6	C57—C56—C55	120.7 (8)
C14—C15—H15	119.6	C57—C56—H56	119.6
C15—C16—C17	119.9 (8)	C55—C56—H56	119.6
C15—C16—H16	120.0	C58—C57—C56	121.1 (9)
C17—C16—H16	120.0	C58—C57—H57	119.5
C18—C17—C16	119.0 (9)	C56—C57—H57	119.5
C18—C17—H17	120.5	C57—C58—C59	118.9 (9)
C16—C17—H17	120.5	C57—C58—H58	120.5
C13—C18—C17	120.5 (9)	C59—C58—H58	120.5
C13—C18—H18	119.8	C60—C59—C58	120.6 (8)
C17—C18—H18	119.8	C60—C59—H59	119.7
C24—C19—C20	117.5 (7)	C58—C59—H59	119.7
C24—C19—P2	121.4 (6)	C59—C60—C55	120.0 (8)
C20—C19—P2	121.0 (6)	C59—C60—H60	120.0
C21—C20—C19	121.1 (8)	C55—C60—H60	120.0
C21—C20—H20	119.4	Cl4—Fe2—Cl5	109.67 (12)
C19—C20—H20	119.4	Cl4—Fe2—Cl3	108.95 (10)
C20—C21—C22	121.0 (8)	Cl5—Fe2—Cl3	110.14 (11)
C20—C21—H21	119.5	Cl4—Fe2—Cl6	110.12 (15)
C22—C21—H21	119.5	Cl5—Fe2—Cl6	110.26 (17)
C23—C22—C21	118.8 (8)	Cl3—Fe2—Cl6	107.67 (10)
C23—C22—H22	120.6	Cl8—C61—Cl7	114.4 (6)
C21—C22—H22	120.6	Cl8—C61—H61A	108.7
C22—C23—C24	119.8 (7)	Cl7—C61—H61A	108.7
C22—C23—H23	120.1	Cl8—C61—H61B	108.7
C24—C23—H23	120.1	Cl7—C61—H61B	108.7
C23—C24—C19	121.7 (8)	H61A—C61—H61B	107.6
C23—C24—H24	119.2	Cl8'—C61'—Cl7'	117 (3)
C19—C24—H24	119.2	Cl8'—C61'—H61C	107.9
C26—C25—C30	118.6 (8)	Cl7'—C61'—H61C	107.9
C26—C25—P2	123.1 (6)	Cl8'—C61'—H61D	107.9
C30—C25—P2	118.2 (6)	Cl7'—C61'—H61D	107.9
C27—C26—C25	120.8 (8)	H61C—C61'—H61D	107.2
C27—C26—H26	119.6	Cl8"—C61"—Cl7"	102 (2)
C25—C26—H26	119.6	Cl8"—C61"—H61E	111.3
C26—C27—C28	118.4 (8)	Cl7"—C61"—H61E	111.3
C26—C27—H27	120.8	Cl8"—C61"—H61F	111.3
C28—C27—H27	120.8	Cl7"—C61"—H61F	111.3
C29—C28—C27	121.2 (9)	H61E—C61"—H61F	109.2
C29—C28—H28	119.4		
			
C7—P1—C1—C2	−37.2 (7)	C37—P3—C31—C36	−145.7 (6)
C13—P1—C1—C2	71.2 (7)	C43—P3—C31—C36	106.0 (7)
Fe1—P1—C1—C2	−163.7 (6)	Fe1—P3—C31—C36	−20.1 (7)
C7—P1—C1—C6	145.8 (6)	C37—P3—C31—C32	38.2 (7)
C13—P1—C1—C6	−105.7 (7)	C43—P3—C31—C32	−70.1 (7)
Fe1—P1—C1—C6	19.3 (7)	Fe1—P3—C31—C32	163.8 (6)
C6—C1—C2—C3	1.5 (12)	C36—C31—C32—C33	0.1 (12)
P1—C1—C2—C3	−175.5 (6)	P3—C31—C32—C33	176.0 (6)
C1—C2—C3—C4	−1.5 (13)	C31—C32—C33—C34	0.0 (13)
C2—C3—C4—C5	0.9 (13)	C32—C33—C34—C35	−0.6 (13)
C3—C4—C5—C6	−0.2 (13)	C33—C34—C35—C36	1.2 (13)
C4—C5—C6—C1	0.2 (12)	C32—C31—C36—C35	0.5 (12)
C4—C5—C6—P2	177.1 (7)	P3—C31—C36—C35	−175.6 (6)
C2—C1—C6—C5	−0.9 (12)	C32—C31—C36—P4	176.2 (6)
P1—C1—C6—C5	176.2 (6)	P3—C31—C36—P4	0.1 (9)
C2—C1—C6—P2	−177.9 (6)	C34—C35—C36—C31	−1.2 (12)
P1—C1—C6—P2	−0.9 (9)	C34—C35—C36—P4	−176.6 (7)
C25—P2—C6—C5	38.1 (8)	C49—P4—C36—C31	−103.3 (7)
C19—P2—C6—C5	−70.8 (7)	C55—P4—C36—C31	146.1 (7)
Fe1—P2—C6—C5	164.8 (6)	Fe1—P4—C36—C31	20.0 (7)
C25—P2—C6—C1	−144.8 (6)	C49—P4—C36—C35	72.2 (7)
C19—P2—C6—C1	106.2 (7)	C55—P4—C36—C35	−38.4 (8)
Fe1—P2—C6—C1	−18.2 (7)	Fe1—P4—C36—C35	−164.6 (6)
C1—P1—C7—C12	128.4 (7)	C43—P3—C37—C38	155.9 (6)
C13—P1—C7—C12	21.4 (8)	C31—P3—C37—C38	48.5 (7)
Fe1—P1—C7—C12	−113.8 (6)	Fe1—P3—C37—C38	−68.9 (7)
C1—P1—C7—C8	−51.5 (7)	C43—P3—C37—C42	−23.3 (8)
C13—P1—C7—C8	−158.6 (6)	C31—P3—C37—C42	−130.7 (7)
Fe1—P1—C7—C8	66.3 (7)	Fe1—P3—C37—C42	111.9 (6)
C12—C7—C8—C9	2.1 (11)	C42—C37—C38—C39	−2.0 (11)
P1—C7—C8—C9	−177.9 (6)	P3—C37—C38—C39	178.8 (6)
C7—C8—C9—C10	−1.2 (12)	C37—C38—C39—C40	0.1 (12)
C8—C9—C10—C11	−0.1 (13)	C38—C39—C40—C41	1.4 (13)
C9—C10—C11—C12	0.6 (13)	C39—C40—C41—C42	−1.0 (13)
C8—C7—C12—C11	−1.6 (12)	C40—C41—C42—C37	−1.0 (13)
P1—C7—C12—C11	178.4 (6)	C38—C37—C42—C41	2.4 (12)
C10—C11—C12—C7	0.3 (13)	P3—C37—C42—C41	−178.3 (6)
C1—P1—C13—C14	−179.5 (6)	C37—P3—C43—C44	77.3 (7)
C7—P1—C13—C14	−74.3 (7)	C31—P3—C43—C44	−177.6 (6)
Fe1—P1—C13—C14	62.2 (7)	Fe1—P3—C43—C44	−58.5 (7)
C1—P1—C13—C18	0.4 (8)	C37—P3—C43—C48	−105.1 (7)
C7—P1—C13—C18	105.7 (7)	C31—P3—C43—C48	−0.1 (8)
Fe1—P1—C13—C18	−117.9 (6)	Fe1—P3—C43—C48	119.0 (6)
C18—C13—C14—C15	0.0 (12)	C48—C43—C44—C45	2.1 (12)
P1—C13—C14—C15	180.0 (6)	P3—C43—C44—C45	179.7 (6)
C13—C14—C15—C16	1.0 (13)	C43—C44—C45—C46	−1.7 (13)
C14—C15—C16—C17	−0.8 (13)	C44—C45—C46—C47	1.3 (13)
C15—C16—C17—C18	−0.4 (13)	C45—C46—C47—C48	−1.4 (14)
C14—C13—C18—C17	−1.2 (13)	C46—C47—C48—C43	1.8 (14)
P1—C13—C18—C17	178.8 (7)	C44—C43—C48—C47	−2.1 (12)
C16—C17—C18—C13	1.4 (13)	P3—C43—C48—C47	−179.7 (7)
C25—P2—C19—C24	66.4 (7)	C36—P4—C49—C50	7.1 (8)
C6—P2—C19—C24	171.3 (6)	C55—P4—C49—C50	113.5 (7)
Fe1—P2—C19—C24	−70.3 (7)	Fe1—P4—C49—C50	−110.5 (7)
C25—P2—C19—C20	−118.1 (7)	C36—P4—C49—C54	−174.0 (6)
C6—P2—C19—C20	−13.3 (8)	C55—P4—C49—C54	−67.6 (7)
Fe1—P2—C19—C20	105.2 (7)	Fe1—P4—C49—C54	68.4 (7)
C24—C19—C20—C21	−1.5 (13)	C54—C49—C50—C51	−0.6 (13)
P2—C19—C20—C21	−177.1 (7)	P4—C49—C50—C51	178.3 (7)
C19—C20—C21—C22	−1.7 (14)	C49—C50—C51—C52	0.7 (14)
C20—C21—C22—C23	2.4 (14)	C50—C51—C52—C53	0.7 (14)
C21—C22—C23—C24	0.0 (12)	C51—C52—C53—C54	−2.3 (13)
C22—C23—C24—C19	−3.3 (12)	C52—C53—C54—C49	2.5 (12)
C20—C19—C24—C23	4.0 (12)	C50—C49—C54—C53	−1.0 (12)
P2—C19—C24—C23	179.6 (6)	P4—C49—C54—C53	−179.9 (6)
C19—P2—C25—C26	−29.2 (8)	C36—P4—C55—C56	139.5 (7)
C6—P2—C25—C26	−135.8 (7)	C49—P4—C55—C56	32.2 (8)
Fe1—P2—C25—C26	105.9 (7)	Fe1—P4—C55—C56	−102.1 (7)
C19—P2—C25—C30	151.7 (7)	C36—P4—C55—C60	−42.9 (7)
C6—P2—C25—C30	45.2 (7)	C49—P4—C55—C60	−150.2 (7)
Fe1—P2—C25—C30	−73.2 (7)	Fe1—P4—C55—C60	75.4 (7)
C30—C25—C26—C27	3.6 (13)	C60—C55—C56—C57	−1.2 (13)
P2—C25—C26—C27	−175.4 (7)	P4—C55—C56—C57	176.3 (7)
C25—C26—C27—C28	−1.8 (14)	C55—C56—C57—C58	0.0 (15)
C26—C27—C28—C29	−1.5 (15)	C56—C57—C58—C59	1.7 (16)
C27—C28—C29—C30	3.0 (16)	C57—C58—C59—C60	−2.2 (15)
C28—C29—C30—C25	−1.1 (14)	C58—C59—C60—C55	1.0 (14)
C26—C25—C30—C29	−2.2 (13)	C56—C55—C60—C59	0.7 (13)
P2—C25—C30—C29	176.9 (7)	P4—C55—C60—C59	−177.0 (7)
